# Development of a DNA Aptamer‐Based Approach to Noninvasively Image CAR‐T Cells In Vivo and Traceless Enrichment In Vitro

**DOI:** 10.1002/advs.202506746

**Published:** 2025-05-11

**Authors:** Minghui Chen, Pengzhao Chang, Zhen Zhang, Dan Liu, Rui Hou, Ming Shi, Jingjing Li, Kai Xu, Junnian Zheng

**Affiliations:** ^1^ School of Medical Imaging Xuzhou Medical University Xuzhou Jiangsu 221004 China; ^2^ Cancer Institute Xuzhou Medical University Xuzhou 221002 China; ^3^ Department of Radiology Affiliated Hospital of Xuzhou Medical University Xuzhou 221006 China; ^4^ Center of Clinical Oncology The Affiliated Hospital of Xuzhou Medical University Xuzhou 221002 China; ^5^ Jiangsu Center for the Collaboration and Innovation of Cancer Biotherapy Xuzhou Medical University Xuzhou 221002 China

**Keywords:** CAR‐T cells, DNA aptamers, enrichment, fluorescence imaging, tracking

## Abstract

Chimeric antigen receptor (CAR) T cells offered a potential cure for malignancies, however, their outcomes and dynamics across different anatomical sites remained inadequately characterized. Monitoring the bio‐distribution and tumor‐homing of CAR‐T cells in vivo is crucial, as it provides patient‐specific data that might inform on treatment success, potential failure, and off‐target toxicities. Herein, an Aptamer A3 by Cell‐SELEX (systematic evolution of ligands by exponential enrichment) is generated, which can bind with CAR‐T cells with nanomolar affinity. After CAR‐T cells are injected into Nalm6 xenograft tumor model mice through tail vein, Cy5‐labeled A3 is injected into mice for fluorescence time‐delay imaging in vivo. The fluorescence signal produced by the Cy5‐labeled A3 is accumulated in the tumor area and reached its maximum at day 14. Moreover, A3 could enrich CAR‐T cells in mixed cell populations in a traceless way. A3 is screened for CAR‐T cells imaging and CAR‐T cells enrichment, which may provide assistance for the evaluation of CAR‐T cells efficacy and the manufacture of CAR‐T cells. Overall, this research shows that A3 enabled repeated, sensitive, and specific assessment of the infused CAR‐T cells in vivo. The screened aptamer will have broad applications for tracking CAR‐T cells in patients, providing insights into treatment success, potential failure, and off‐target toxicities.

## Graphical abstract

A DNA aptamer (A3) with nanomolar affinity for CAR‐T cells is developed via Cell‐SELEX. In Nalm6 tumor‐bearing mice, Cy5‐labeled A3 enables long‐term fluorescence imaging and tracks the biodistribution of CAR‐T cells, with tumor signal peaking on day 14 after CAR‐T cell administration. Additionally, A3 facilitates traceless enrichment of CAR‐T cells from mixed populations, thereby enhancing efficacy evaluation and manufacturing processes.

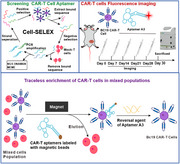

## 1 Introduction

Chimeric antigen receptor (CAR) T cell therapy has shown a promising curative strategy for multiple haematological malignancies.^[^ ^1^ ^]^ T‐cell surfaces were modified with synthetic CARs to target tumors and eliminated cancer cells. CAR‐T cells targeting CD19 had shown effective for the treatment of B‐cell malignancies.^[^ ^2^ ^]^ Currently, the US Food and Drug Administration (FDA) had approved four CAR19‐T cell therapies for patients with B‐cell malignant diseases.^[^ ^3^ ^]^ CAR‐T cell therapies were heralded as the third revolution in the biomedical industry following small molecule and antibody drugs.^[^ ^4^ ^]^ Despite these promising results, the responses in patients undergoing CAR‐T cell therapies had been inconsistent.^[^ ^5^ ^]^ Different from conventional drugs, which adhered to the traditional pharmacological principles of absorption, distribution, metabolism, and excretion, CAR‐T cells as a “living” therapeutic agent posed challenges in evaluating their pharmacokinetic characteristics. Once administered to the patient, CAR‐T cell therapies entered a “black box”, which had been a significant obstacle to the development of CAR‐T therapy. Due to the lack of effective monitoring of CAR‐T cells drugs in vivo, the reasons for many cancer patients who did not respond to CAR‐T cells therapy or experienced subsequent relapse are still unknown and cannot be predicted.^[^ ^6^ ^]^ Therefore, a diagnostic approach capable of tracking the distribution and proliferation of CAR‐T cells in tumors and off‐target locations will improve personalized treatment strategies and combination therapy regimens.

Clinical investigators utilized various strategies to assess the functionality of infused CAR‐T cells, such as flow cytometry, immunohistochemistry, and quantitative polymerase chain reaction (qPCR) analysis of peripheral blood samples or biopsies from sites of active CAR‐T cell infiltration such as bone marrow, tumors, and lymph nodes.^[^ ^7^ ^]^ However, repeated invasive biopsies were often impractical in numerous clinical trials.^[^ ^8^ ^]^ Therefore, imaging the distribution process of CAR‐T cells after administration and analyzing their distribution and homing kinetics in vivo would significantly aid in proactively assessing tumor treatment efficacy.^[^ ^9^ ^]^

The in vivo tracking way of CAR‐T cells could be divided into indirect‐labeling and direct‐labeling methods.^[^ ^7^ ^]^ Indirect‐labeling methods enabled observation over the entire lifetime of CAR‐T cells, facilitating long‐term tracking of their biological distribution in vivo. This was commonly achieved by introducing a reporter gene into the CAR‐T cells, which was translated into reporter proteins if their expressions were stable.^[^ ^10^ ^]^ However, extra imaging reporter gene into the CAR‐T cells might affect the persistence and immune function of CAR‐T cells. Common drawbacks of indirect‐labeling method further include the complexity of design and operation, promoter silencing, and the potential for DNA modification‐induced mutations.^[^ ^11^ ^]^ Direct‐labeling methods were a simple process that eliminated the operation for genetic modification of the CAR‐T cells. CAR‐T cells actively incorporated labels during in vitro culture, and subsequently entered in vivo together via injection.^[^ ^12^ ^]^ Unfortunately, this approach was limited by its time resolution for in vivo CAR‐T cells observation, as the marker was diluted or lost during CAR‐T cell proliferation, thereby presenting less and less tracer signal.^[^ ^13^ ^]^ To fabricate imaging probe which can be repeated administrated to track naturally expressed genes or proteins in CAR‐T cells might mitigate label dilution or loss during cell division. Thus, the choice of a suitable binding molecule is essential to realize this aim. The development of in vivo indirect‐labeling long‐term imaging methods that avoid genetic modification of CAR‐T cells has become a significant research trend, as it does not require altering the established protocols for CAR‐T cell therapy.^[^ ^14^ ^]^ Although the inducible T‐cell costimulator (ICOS) has been considered as a potential target antigen for antibody‐based CAR‐T cell imaging,^[^ ^14a^ ^]^ its utility is limited by an inability to reliably detect resting‐state CAR‐T cells following activation, which is a critical capability for evaluating their long‐term persistence. Mohammad Rashidian's group developed an antigen‐based imaging strategy using CD19 as a probe, enabling specific binding to CD19 CAR‐T cells through high‐specificity recognition of the surface‐expressed CAR protein.^[^ ^14b^ ^]^ However, antibodies or antigens, owing to their larger molecular weight and slower clearance rates, are more likely to accumulate on the surface of CAR‐T cells, potentially affecting the efficacy of CAR‐T cell therapy.

Aptamers are single‐stranded oligonucleotides that can specifically bind to target molecules and have demonstrated their potential for in vivo cell tracking owing to advantages such as low molecular weight, high programmability, and rapid in vivo clearance.^[^ ^15^ ^]^ Aptamers can fold into a 3D conformation to specifically bind to a wide range of targets, including proteins, peptides, small molecules, and metal ions. The majority of aptamers were discovered through a library selection process called systematic evolution of ligands by exponential enrichment (SELEX), which enriches binding sequences from a large pool of random oligonucleotides using a target of interest.^[^ ^16^ ^]^ Aptamers were often referred as “chemical antibodies”. But compared to antibodies, they were easier to store, cheaper to produce. They could also be chemically synthesized and penetrated tissue quickly.^[^ ^17^ ^]^ Previous experiments had also proved the metabolism and distribution of aptamers in the human body, which possessed clinical safety.^[^ ^18^ ^]^ Thus, aptamers were produced as well‐defined, low‐variability products with long storage stability, making them cost‐effective and safe for specific and sensitive recognizing of CAR‐T cells, thereby enabling efficient in vivo tracking of infused CAR‐T cells.

Here, we obtained an Aptamer A3 with specific binding ability to CAR‐T cells through Cell‐SELEX technology and further applied it to track the distribution of CAR‐T cells in vivo. Bispecific BC19 CAR‐T cells,^[^ ^19^ ^]^ which can target tumor cells overexpressing BCMA (B Cell Maturation Antigen) and CD19 (Cluster of Differentiation 19) antigens both in vitro and in vivo, were fabricated and utilized as the target cells for this research. During positive screening, single‐stranded DNA (ssDNA) random library was incubated with CAR‐T cells. Thereafter, cell‐bound oligonucleotides were extracted and negative selection was performed, using non‐target cells (Mock‐T cells) to remove non‐specifically bound oligonucleotides. The liquid supernatant containing ssDNA with affinity to CAR‐T cells was collected for PCR amplification. After screening process of ≈11 cycles, the final library reached a certain level of significant affinity and was then sequenced. After the aptamer sequences was determined, CAR‐T cells were monitored in vivo for one month by direct labeling with Cy5‐labeled aptamer, demonstrating the potential of our selected aptamer as molecular imaging probe for CAR‐T cells expansion and bio‐distribution. In addition, we used selected aptamer to bind its complementary reverse DNA sequences to enrich CAR‐positive cells in a trace‐free manner. Totally in all, a novel CAR‐T cell aptamer had been identified that provided a highly efficient, accessible, and cost‐effective alternative tool for enriching, monitoring, and targeting CAR‐T cells in potential clinical‐scale cell therapy applications.

## 2 Results and Discussion

### 2.1 Identification of CAR‐T Cells Binding Aptamers by Cell‐SELEX

**Figure 1:**
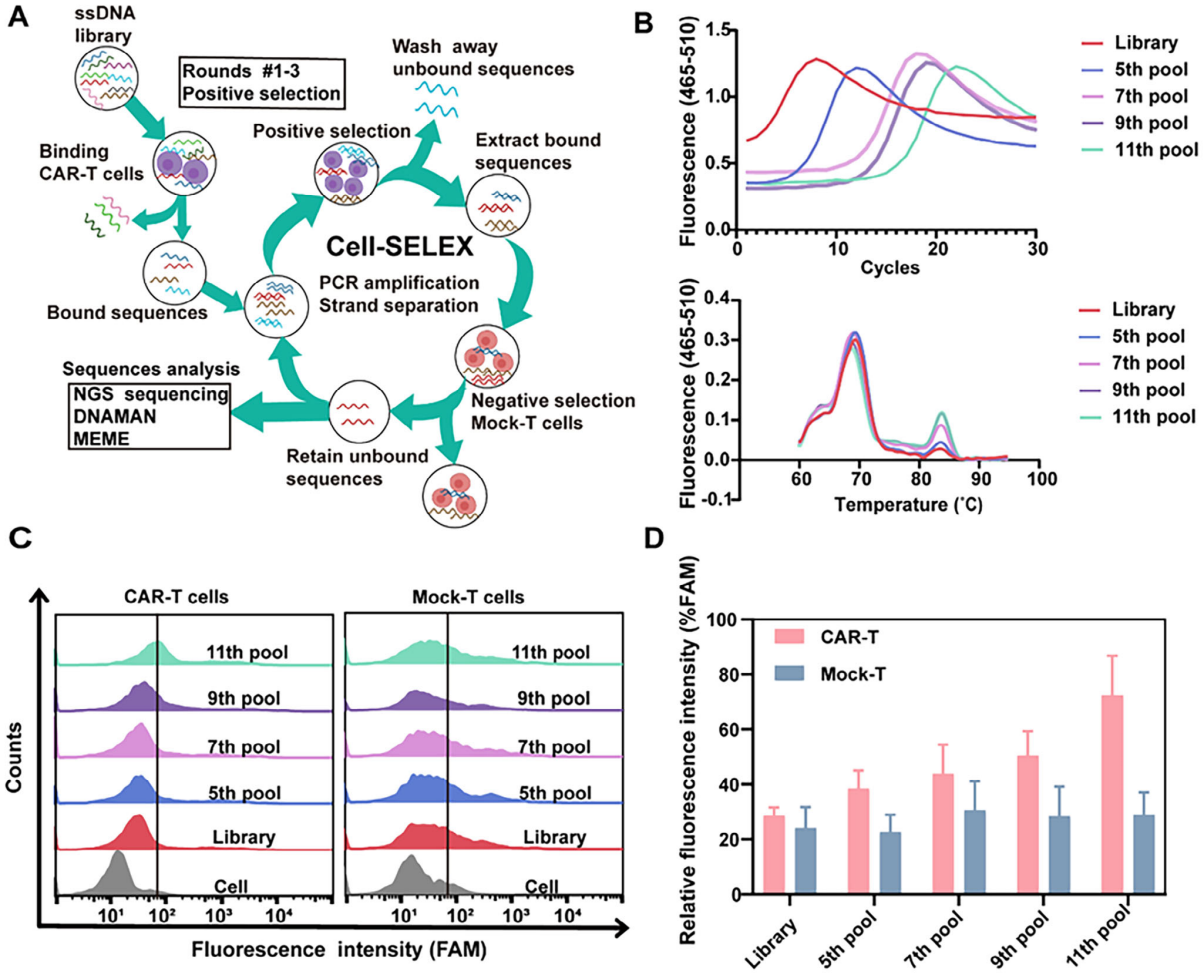
Aptamer screening process for CAR‐T cells. A) Schematic illustration of Cell‐SELEX using CAR‐T cells for positive selection and Mock‐T cells for negative selection. B) AC (upper panel) and MC (lower panel) of the elution solution from different rounds of the ssDNA pool during CAR‐T cells aptamers selection. Reaction volumes (20 µL) containing 300 nmol primers, 2×SYBR GREEN qPCRmix, and DNA template on Light Cycler Real‐Time PCR System (Roche 480). C) The binding performance of aptamers, ssDNA library, 5th pool, 7th pool, 9th pool, and 11th pool to CAR‐T cells and Mock‐T cells by flow cytometry. D) Quantitative analysis of fluorescence intensity in enriched DNA pools during different selection rounds for CAR‐T cells and Mock‐T cells, data were obtained from flow cytometry. Error bars represented the standard deviation (n=3).

To develop specific DNA aptamers targeting CAR‐T cells, bispecific CAR‐T cells,^[^ ^19^ ^]^ targeting BCMA/CD19 were selected as target for positive selection. Mock‐T cells were employed for negative selection. The slightly modified Cell‐SELEX procedure was schematically depicted in **Figure** **1**A. After three rounds of positive selection against CAR‐T cells with single‐strand DNA library containing 36 nucleotides random region, the collected aptamers underwent negative selection by incubation with Mock‐T cells to eliminate nonspecific sequences. As shown in Figure S1A,B (Supporting Information), evolutionary library of each round was analyzed by qPCR to determine the optimum number of cycles for preparing PCR.^[^ ^20^ ^]^ To increase the yield of ssDNA products, six replicates of the CAR‐T cell‐SELEX experiments were performed simultaneously. The six curves in Figure S1B (Supporting Information) depicted the fluorescence qPCR results of the 1th round pool derived from these six independent replicates Cell‐SELEX experiments. The optimal number of PCR cycles was 20 cycles on the basis of the maximum number of cycles of the amplification curve of the aptamer evolution pool (Table S1, Supporting Information). Single‐strand DNA (ssDNA) separation from PCR products was achieved through polyacrylamide gel electrophoresis. Each well of the gel corresponds to identical products, representing ssDNA preparations derived from the PCR product of the 1st round library, as shown in Figure S1C,D (Supporting Information). To monitor the degree of enrichment, the ssDNA libraries were analyzed at the 0st, 5th, 7th, 9th, and 11th selection rounds using qPCR amplification curves (AC), melting curves (MC), and flow cytometry. (Figure 1B,C). The ACs changed along with the selection conduct (Figure 1B, upper panel). The progressive increase in cycle threshold (Ct) values and delayed fluorescence rise in later SELEX rounds (e.g., 11th round vs. library) reflect reduced ssDNA concentration in the elution pool, demonstrating successful depletion of non‐binding aptamers and enrichment of high‐affinity candidates through iterative selection. Simultaneously, the corresponding MC exhibited a clear double peaks evolutionary process (Figure 1B, lower panel), indicating a change in the diversity of the ssDNA library.^[^ ^20^ ^]^ Flow cytometry results showed that as the cycles of enriched DNA pools increased, the fluorescence intensity of CAR‐T cells progressively elevated (Figure 1C). The fluorescence intensities at the two black vertical lines in Figure 1C were identical. These lines were added to emphasize the enhanced fluorescence enrichment of CAR‐T cells in the evolution pool library compared to the random library. By comparison, Mock‐T cells exhibited minimal fluorescence intensity, similar to the levels observed in the control group (Figure 1D). Additionally, flow cytometry revealed no significant signal difference between the 11th pool and 12th pool, which prompted us to terminate the selection process (Figure S2, Supporting Information).

The 11th round aptamer pool sequences were then identified by next generation sequencing (NGS). DNAMAN ranks and compares aptamer sequences based on their frequency, while the Multiple Em for Motif Elicitation (MEME) Suite groups aptamers according to their motifs.^[^ ^21^ ^]^ In an ideal selection process, the binding sequences are those with the highest frequency and the most prevalent motif.^[^ ^22^ ^]^ As shown in Figure S3A (Supporting Information), the sequences highlighted in pink have a high degree of homology. The evolutionary tree visualizes these 22 highly homologous sequences (Figure S3B, Supporting Information). These 22 sequences were analyzed and their binding motifs were predicted using MEME Suite. As shown in Figure S3C,D (Supporting Information), three binding motifs were predicted and motif 1 was common in these highly homologous sequences. Based on sequential homogeneity, predicted motif and repeatability, we chose the sequences with the highest abundance from each family. Consequently, eight candidate aptamers (A1‐A8) were chosen for CAR‐T cells binding studies further.

### 2.2 Characterization of CAR‐T Cells Binding Aptamers

**Figure 2:**
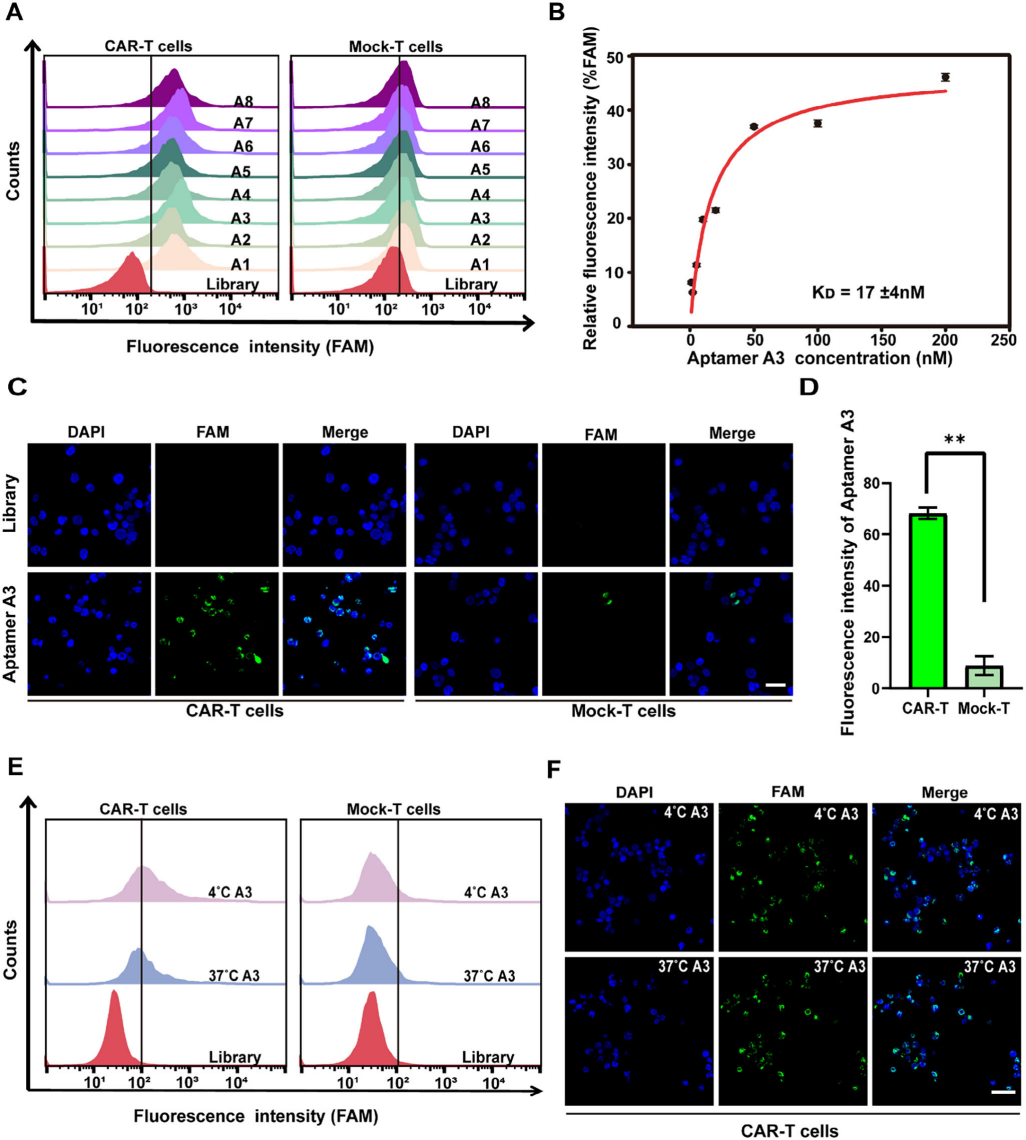
Characterization of candidate aptamers. A) Flow cytometry assessment of the binding efficiency of eight candidate sequences (250 nmol L^−1^) with to target CAR‐T cells and control Mock‐T cells. B) Binding curve of Aptamer A3 to CAR‐T cells by flow cytometry. C) Confocal images of CAR‐T cells and Mock‐T cells stained with DAPI (blue) and co‐cultured with Aptamer A3 (green). Blue and green signals indicate DAPI and FAM‐labeled Aptamer A3, respectively. Scale bar: 20 µm. D) Quantitative analysis of average fluorescence intensities in Confocal images of CAR‐T cells and Mock‐T cells after incubation with the Aptamer A3. E) Flow cytometry analysis of the binding ability of Aptamer A3 with target CAR‐T cells and control Mock‐T cells at 4 °C and 37 °C. F) Confocal images of CAR‐T cells after incubation with Aptamer A3 at 4 °C and 37 °C. Blue and green signals indicate DAPI and FAM‐labeled Aptamer A3, respectively. Scale bar: 20 µm. Error bars represented the standard deviation (n=3). ^*^ *p* < 0.05 and ^**^ *p* < 0.01.

In order to select the best aptamer from the candidates, eight aptamers (named A1, A2, A3, A4, A5, A6, A7, A8) were chemically synthesized (Table S2, Supporting Information). Regarding the cell‐binding specificity of the selected aptamers, the outcome of flow cytometry analysis indicated that the eight aptamers could hardly bind to other cells such as Nalm6, U266, Jurkat, Raw264.7, MAEC, GL‐261 cells (Figure S4, Supporting Information). Furthermore, the eight aptamer candidates exhibited eminent binding ability against CAR‐T cells, whereas no significant binding was observed in Mock‐T cells (**Figure** **2**A), illustrating the feasibility of our selected candidate aptamers to target CAR‐T cells. To further assess the binding ability of candidate aptamers to CAR‐T cells, the apparent K_D_ values of aptamers (A1, A2, A3, A4, A5, A6, A7, A8) were evaluated using flow cytometry. As shown in Figure S5 (Supporting Information) and Figure 2B, the A1, A2, A3, A4, A5, A6, A7 and A8 aptamer exhibited apparent K_D_ values of 13 ± 5, 62 ± 31.3, 17 ± 4, 12 ± 4, 12 ± 3, 10 ± 3, 15 ± 7, and 15 ± 7.5 nM, respectively. Considering the K_D_ value of aptamer A2 was significantly higher than those of other candidate aptamers, we chose the final aptamer from A1, A3, A4, A5, A6, A7, and A8. Then, cellular fluorescence imaging was introduced to further evaluate the binding ability of the selected candidate aptamers to CAR‐T cells intuitively. As depicted in Figure S6A,B (Supporting Information), the fluorescence signal intensity of the interaction between Aptamer A3 and CAR‐T cells was the highest among all aptamer candidates. Consequently, we further evaluated the binding specificity of A3 by comparing its interactions with CAR‐T cells and mock‐T cells (Figure 2C,D), at the same treated condition, Aptamer A3 pronounced a stronger green fluorescence in CAR‐T cells, but less signals in Mock‐T cells, suggesting that Aptamer A3 may be the best candidate for binding to CAR‐T cells. To exclude any potential impact of Fam as a fluorescent labeling dye on the experimental outcomes, we conducted supplementary experiments using Aptamer A3 labeled with Cy5. As shown in Figure S7 (Supporting Information), the results confirmed that the signals observed on the cell membrane surface corresponded to genuine aptamer binding reactions, rather than being resulting from endosomal entrapment or pH‐induced changes. Flow cytometry was used to assess the binding specificity of Aptamer A3 at various concentrations across six different cell types: Nalm6, U266, Jurkat, RAW264.7, MAEC, and GL‐261 cells. As shown in Figure S4G,H (Supporting Information), Aptamer A3 did not bind to other cell types even at higher concentrations. Given that the aptamer was administered to mice at a dose of 4.5 nmol, resulting in an estimated in vivo circulating concentration of ≈2000 nmol L^−1^. These results indicated that Aptamer A3 was well‐suited for in vivo imaging of CAR‐T cells while maintaining high specificity and minimal non‐specific binding to other cells. Considering all of the above factors, Aptamer A3 was chosen for binding to CAR‐T cells. To further evaluate the target recognition performance of Aptamer A3 at in vivo condition, flow cytometry and confocal laser scanning microscope (CLSM) CLSM were performed at 37 °C. As illustrated in Figure 2E,F, both flow cytometry and CLSM results showed that there was no significant difference on the binding ability of Aptamer A3 to CAR‐T cells at 4 °C and 37 °C. Meanwhile, no obvious binding of Aptamer A3 to Mock‐T cells could be observed at either 4 °C or 37 °C. It was clear that Aptamer A3 could bind with CAR‐T cells specifically and possessed the potential for CAR‐T cells monitoring in vivo at 37 °C.

### 2.3 Confirmation of the Aptamer A3 Target Protein

**Figure 3:**
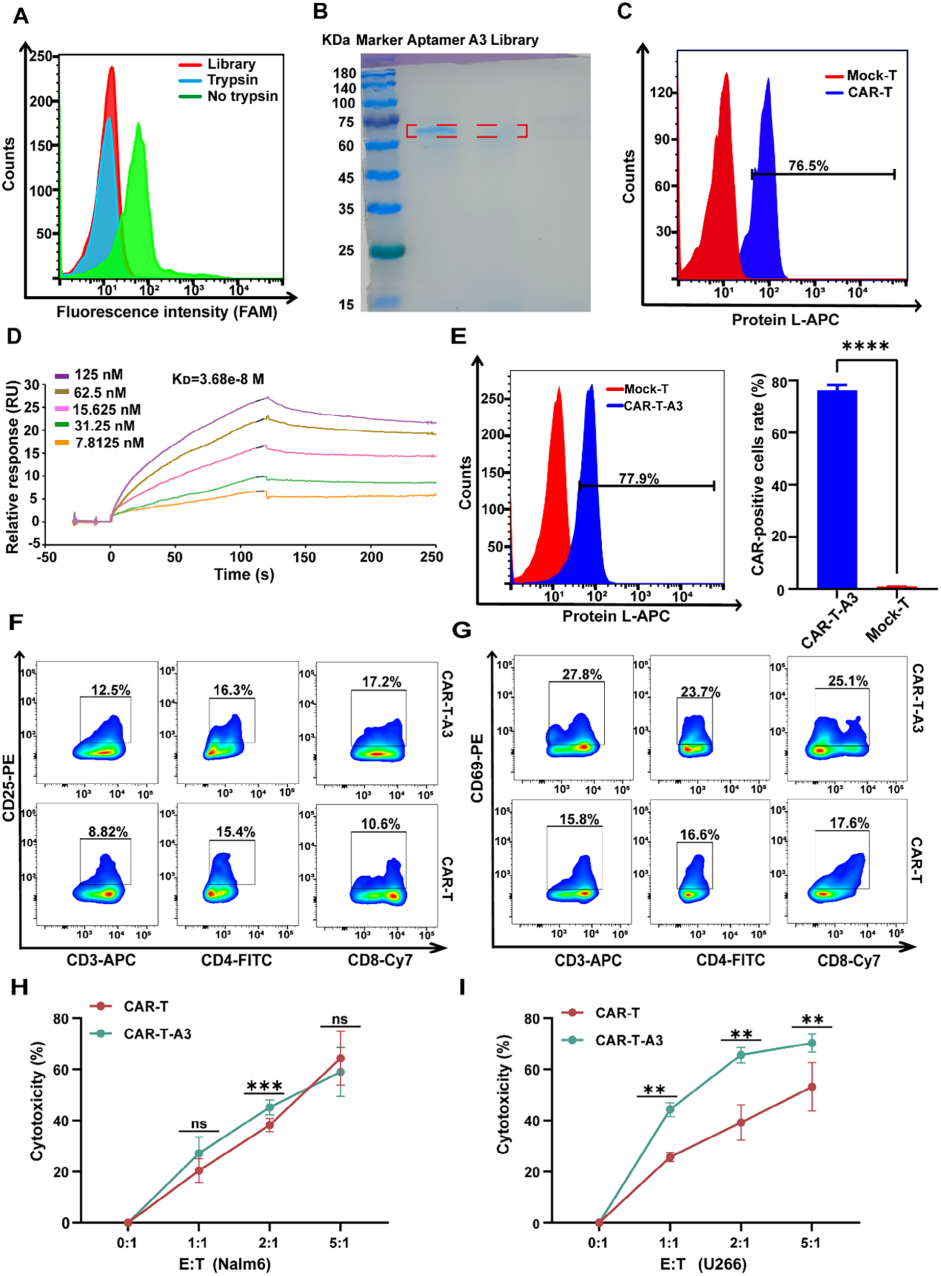
Confirmation of the Aptamer A3 target protein and characterization of CAR‐T cells in vitro after incubation with Aptamers A3. A) After trypsin treatment, the binding of Aptamer A3 to CAR‐T cells was analyzed by flow cytometry. B) Coomassie Brilliant Blue stained 10% SDS‐PAGE was used to evaluate the protein binding to Aptamer A3 (Full‐size image of gel was shown in Figure S11, Supporting Information). C) Flow cytometry analysis of CAR expression on CAR‐T cells incubation without Aptamer A3. D) SPR results of Aptamer A3 for BCMA‐CD19 His protein affinity. E) Flow cytometry analysis of CAR expression on CAR‐T cells‐Aptamer A3 and a statistical graph of the CAR‐positive rate, defined as the percentage of T cells that were positive for protein L binding. F,G) The expression of activation markers CD25 and CD69 in gated CD3+, CD4+, and CD8+ T cell populations from CAR‐T cells and CAR‐T cells‐Aptamer A3. H,I) Cytotoxicity of CAR‐T cells and CAR‐T cells‐Aptamer A3 against Nalm6 and U266 tumor cells. Error bars represented the standard deviation (n=3). ^*^ *p* < 0.05, ^**^ *p* < 0.01, ^***^ *p* < 0.001, and ^****^ *p* < 0.0001.

For Cell‐SELEX screening, any molecule on the surface of the cells might be a potential target. To investigate the type of target molecule bound by Aptamer A3, CAR‐T cells were treated with trypsin and then incubated with Aptamer A3. Flow cytometry results showed that compared with the non‐treated group, Aptamer A3 had no binding ability on CAR‐T cells treated with trypsin (**Figure** **3**A), suggesting that the target molecule of Aptamer A3 was a cell membrane protein. Next, we incubated the biotin‐tagged Aptamer A3 with CAR‐T cells and the target proteins were collected with streptavidin agarose for SDS‐PAGE. Intriguingly, there was a protein band with a relative molecular weight of 60–75 KDa in the Aptamer A3 column, but not in the random library (Figure 3B). Considering the molecular weight of the BCMA‐CD19‐His protein is ≈60 KDa and BC19 CAR expression rate of the target CAR‐T cells we used for Cell‐SELEX was ≈76.5% (Figure 3C), we hypothesized that the aptamers may specifically bind to the bispecific BC19 CAR, which comprises an anti‐BCMA single‐chain variable fragment (scFv), a humanized anti‐CD19 scFv, and a hinge region. This BC19 CAR construct is not expressed on mock‐T cell. To confirm the interaction between aptamers and the BCMA‐CD19‐His fusion protein, we measured their affinity and kinetics by surface plasmon resonance (SPR). The binding capacity of Aptamer A3 with a serial dilution against the BCMA‐CD19‐His protein coupled to the CM5 chip was monitored. As illustrated in Figure 3D, the Aptamer A3 had the ability to bind with BCMA‐CD19‐His protein with a binding affinity (K_D_ value) of 37 nM, which was comparable with their binding ability to CAR‐T cells (17 ± 4 nM). Meanwhile, as can be seen from Figure S8 (Supporting Information), compared with the SPR signals presented in Figure 3D for aptamer A3 binding to BCMA‐CD19‐His protein complex, aptamer A3 exhibited negligible binding affinity toward His protein, BCMA protein, and CD19 protein individually. The above data suggested that the complete protein complex of BCMA‐CD19‐His was the potential binding target of Aptamer A3. Therefore, the aptamer A3 is specifically applicable for imaging BC19 CAR‐T cells.

### 2.4 Functionality Study of CAR‐T Cells After Binding with Aptamer A3 In Vitro

CAR‐T cells are activated to eliminate tumor cells after binding to the targeted antigen. It is vital to confirm that the binding of Aptamer A3 to CAR‐T cells will not affect their therapeutic function. For CAR‐T cells, the effective components that played a role in tumor killing were CAR‐positive T cells. Thus, CAR expression rate of CAR‐T cells after binding with Aptamer A3 (250 nmol L^−1^) was first detected by a flow cytometry system. As illustrated in Figure 3C,E, CAR expression rates on CAR‐T cells without aptamer incubation was 76.5% and ≈77.9% with Aptamer A3 binding (CAR‐T cells‐Aptamer A3), indicating that Aptamer A3 binding did not affect the expression of CAR in CAR‐T cells. CD25 and CD69 are up‐regulated through TCR (T cell receptor) signal transduction and are commonly used as markers for T cell activation. The expressions of CD25 and CD69 in CAR‐T cells were tested with or without aptamer A3 (250 nmol L^−1^) co‐incubation by flow cytometry. As depicted in Figure 3F,G and Figure S9A,B (Supporting Information), obvious difference was observed on both CD25 and CD69 expressions in CAR‐T cells and CAR‐T cells‐Aptamer A3. These results may be attributed to the DNA aptamers mimicking the function of an immune adjuvant, thereby influencing the activation of CAR‐T cells.^[^ ^23^ ^]^ To investigate whether such features are aptamer concentration‐dependent, Flow Cytometry analysis was performed to detect CAR expression rates and the expressions of CD25 and CD69 in CAR‐T cells after binding with higher concentrations of aptamer A3 (500 and 2000 nmol L^−1^). As shown in Figure S9C–F (Supporting Information), neither CAR expression (Figure S9C,D, Supporting Information) nor CD25/CD69 (Figure S9E,F, Supporting Information) levels showed significant variations across the tested concentrations of Aptamer A3, indicating that these features are independent of aptamer concentration. Finally, the effective killer functions of CAR‐T cells and CAR‐T cells‐Aptamer A3 on tumor cells were further compared in vitro by LDH (lactate dehydrogenase) assay. CAR‐T cells‐Aptamers A3 exhibited a comparable and even better cytolytic capacity against Nalm6 and U266 malignant cells in an E:T ratio‐dependent manner (Figure 3H,I). These results demonstrated the function of CAR‐T cells was not affected by the combination of Aptamer A3 and CAR‐T cells. To further evaluate whether the aptamer modification impacts the anti‐tumor efficacy of CAR‐T cells, the in vivo experiments was conducted. As shown in Figure S10A–C (Supporting Information), the tumor volume in the PBS control group reached ≈600 mm^3^ at day 12 post‐treatment. In contrast, both the CAR‐T cells group and the CAR‐T cells‐Aptamer A3 group exhibited significantly suppressed tumor growth, with tumor volumes increasing only to ≈300 mm^3^. These results demonstrated that aptamer conjugation does not compromise the therapeutic efficacy of CAR‐T cells and effectively inhibits the progression of NALM‐6 tumors. Notably, no significant fluctuations in mouse body weight were observed throughout the treatment period, indicating that the aptamer‐modified CAR‐T cells exhibited favorable biosafety profiles (Figure S10D, Supporting Information).

### 2.5 Stability of the Aptamers in Serum

**Figure 4:**
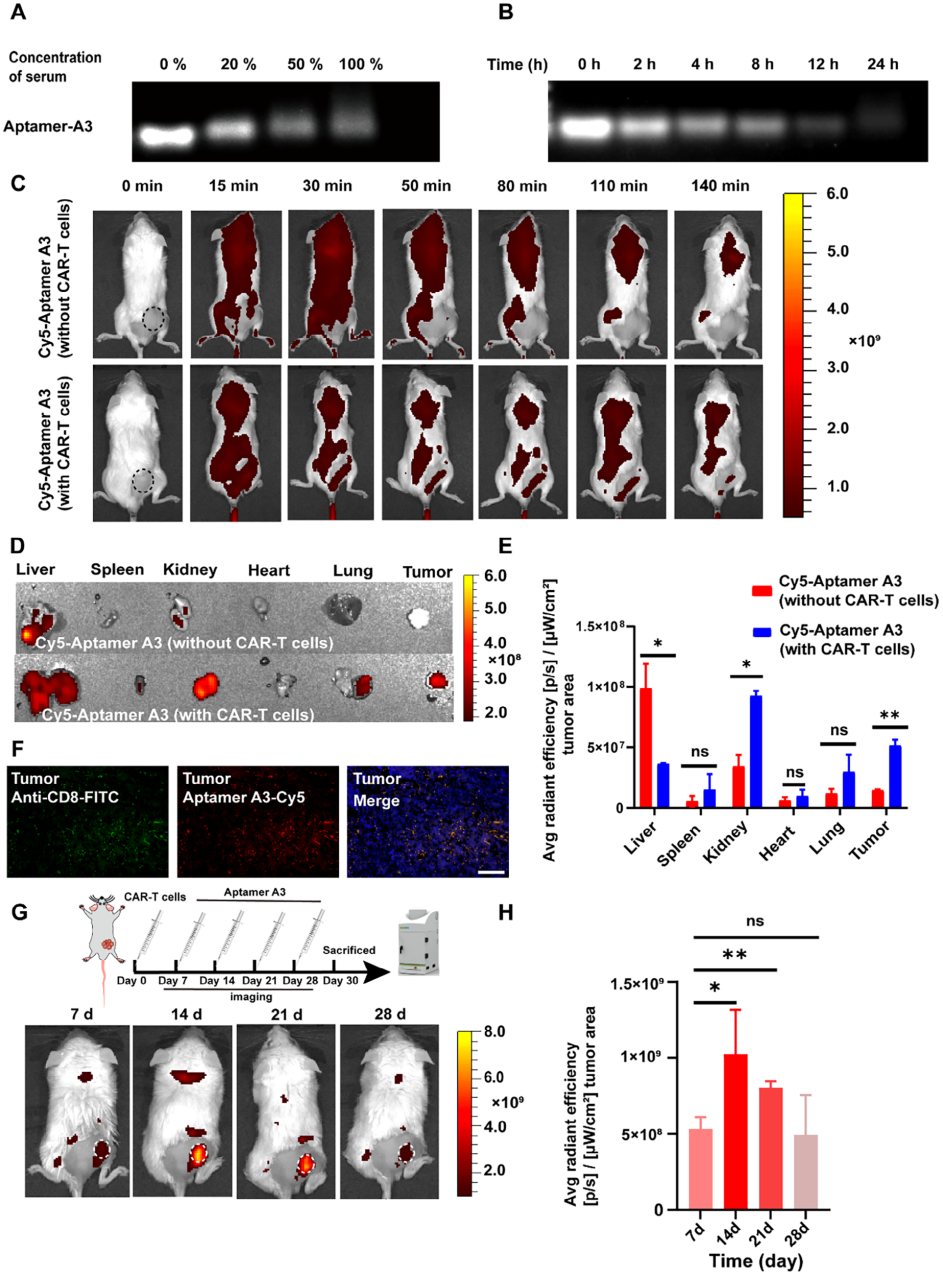
Whole‐body fluorescence in vivo images of mouse xenogeneic tumor model bearing Nalm6 tumor injected by CAR‐T cells taking direct labeling strategies (n = 3 per group). Agarose gel electrophoresis to confirmed its residual products of Aptamer A3 incubated A) in human serum with different concentrations (0%, 20%, 50%, 100%) for 2h and B) in 20% human serum at the indicated time points (0, 2, 4, 8, 12, and 24 h). (Full‐size images of gel are shown in Figure S13A,B, Supporting Information). C) Images of tail vein injection of Cy5‐labeled Aptamer A3 into mice without CAR T cells and into mice with CAR‐T cells. D) Ex vivo organ fluorescent images of Cy5‐labeled Aptamer A3 into mice without CAR‐T cells and into mice with CAR‐T cells. E) Quantitative analysis of average fluorescence intensities in major organs and tumor tissues. F) Immunofluorescence‐staining images of tumor tissues after Cy5‐labeled Aptamer A3 injection. Blue channel: DAPI; Red channel: Aptamer A3, Green channel: Anti‐CD8‐FITC. The scale bar indicates 100 µm. G) In vivo fluorescence imaging of mice injected CAR‐T cells was performed for one month using Aptamer A3. Fluorescence image acquisition for each time period was carried out half an hour after the Cy5‐labeled Aptamer A3 injection. H) Quantified of average fluorescence intensities in tumor area based on the fluorescence images in different time. Error bars represented the standard deviation (n=3). ^*^ *p* < 0.05 and ^**^ *p* < 0.01.

The excellent behaviors of Aptamer A3 in vitro stimulated us to look for the possibility of their use in CAR‐T cells tracking in vivo. The stability of aptamers in blood circulation is critical for successful in vivo imaging applications. Thus, the stability of Aptamer A3 was initially assessed by incubating it in human serum at 37 °C for varying concentrations and time periods. In the physical environment, the proportion of serum to total blood volume was 20%–40%. We set serum concentrations of 0%, 20%, 50%, and 100% to test the stability of Aptamer A3 in vitro. As displayed in **Figure** **4**A, although the intensity of Aptamer A3 bands gradually dimmed with the increase of serum concentration, the band remained discernible even at 100% serum concentration. With 20% serum concentration, Aptamer A3 could still be remained discernible for 24 h incubation, which is sufficient and favorable for in vivo monitoring for Aptamer A3 (Figure 4B). Previous studies had also suggested that the presence of serum in vivo had little effect on target detection of high‐affinity aptamers,^[^ ^24^ ^]^ further strengthening the possibility of Aptamer A3 based CAR‐T cell tracking in vivo.

### 2.6 In Vivo Fluorescence Tracking of CAR‐T Cells by Aptamer A3 in Nalm6 Tumor‐Bearing Mice Models

To verify the CAR‐T cells tracking by Aptamer A3 in vivo, a xenograft tumor mouse model was developed where the Nalm6 cells were implanted subcutaneously to the right lateral thigh. 2 × 10^6^ CAR‐T cells were first injected intravenously into Nalm6 tumor‐bearing mice. Twelve hours later, Cy5‐labeled Aptamer A3 was injected into the mouse through the tail vein. Based on the stability of Aptamer A3 in serum, the fluorescence signal collection time was set from 15 to 140 min. The model mouse without CAR‐T cells injection were employed as control. As shown in Figure 4C, the fluorescence signal in control group was uniformly distributed initially, but no substantial fluorescence signal could be discerned in the tumor region within 140 min post‐injection period with Aptamer A3. But for the group with CAR‐T cells injection, the fluorescence signal was distributed throughout the tumor mouse model analogously within the first 30 min after Cy5‐labeled Aptamer A3 injection, and such fluorescence signals in the tumor area were more contrast at 50 min, 80 min and 110 min, which became weak until 140 min, possibly due to the filtering of the aptamer by the kidney. Furthermore, no significant fluorescence was detected in the tumor region and the dissected tumor after the injection of Cy5‐labeled random sequences in a tumor mouse model with CAR‐T cells (Figure S12A,B, Supporting Information). The intensities of Cy5 fluorescence from major organs (liver, spleen, kidney, heart, lung) were also detected. Compared with mouse that did not receive CAR‐T cell injection, within 140 min after CAR‐T cell administration, a subset of Cy5‐labeled Aptamer A3 redistributed from the liver to the kidneys and tumor within 140 min after CAR‐T administration. Such difference is likely related to the metabolic pathways of CAR‐T cells. Some CAR‐T cells potentially being cleared via renal pathways, which is consistent with prior findings regarding the elimination mechanisms of cell therapies.^[^ ^14b^ ^]^ Additionally, stronger fluorescence signals of Aptamer A3 were detected in the tumor regions of mouse injected with CAR‐T cells, illustrating Aptamer A3 has outstanding specificity for CAR‐T cells in tumor region (Figure 4D,E).

To further testify the fluorescence signal from Aptamer A3 matching the location of CAR‐T cells, co‐localization of CD8 antibody and Aptamer A3 was evaluated because CD8+ T cells were the main cells that mediate antigen‐specific immune responses in CAR‐T cells therapy. As illustrated in Figure 4F, the green color from CD8 antibody and the red emission from Aptamer A3 were merged in tumor tissue sections, illustrating Aptamer A3 had the ability to track CAR‐T cells in vivo, which made it a promising tool for monitoring CAR‐T cell tumor‐homing efficiency in vivo. To investigate the continuous monitoring of CAR‐T cells by Aptamer A3, the CAR‐T cells were tracked for a month in vivo by a slightly variable direct labeling strategy. As shown in Figure 4G, CAR‐T cells (2 × 10^6^) were injected intravenously into Nalm6 tumor‐bearing mice on day 0. Then, the mice were injected intravenously with Cy5‐labeled Aptamer A3 and underwent fluorescence imaging in vivo on days 7, 14, 21, and 28, respectively. Fluorescence signal acquisition was consistently performed 30 min post‐injection of Cy5‐labeled Aptamer A3. As illustrated in Figure 4H, the intensity of accumulated fluorescence signal from Aptamer A3 was 5.04 × 10^8^ [p/s/cm^2^/sr]/[µW/cm^2^] at day 7 post CAR‐T cells administration, and gradually increased and escalated to its zenith at day 14 (1.02 × 10^9^ [p/s/cm^2^/sr]/[µW/cm^2^]), persisting until day 21 (7.36 × 10^8^ [p/s/cm^2^/sr]/[µW/cm^2^]), which was declined to be 4.94 × 10^8^ [p/s/cm^2^/sr]/[µW/cm^2^] at day 28. Strong fluorescence was also observed in the dissected tumor on day 14 (Figure S14, Supporting Information), which was consistent with the CAR‐T cell proliferation rule in tumor region.^[^ ^25^ ^]^ Moreover, our previous research has also demonstrated that, BC19 CAR‐T cell amplification in peripheral blood peaked at a median of 12 days (range, 4–25) after infusion followed by a gradual decrease.^[^ ^19^ ^]^ These data demonstrated that CAR‐T cells could be continuously monitored in vivo by Aptamer A3 over a long period of time, and suggested that Aptamer A3 has the potential to serve as a tool for obtaining real‐time distribution and persistence information of CAR‐T cells in vivo.

### 2.7 Traceless Enrichment of CAR‐T Cells Using Aptamer A3

**Figure 5:**
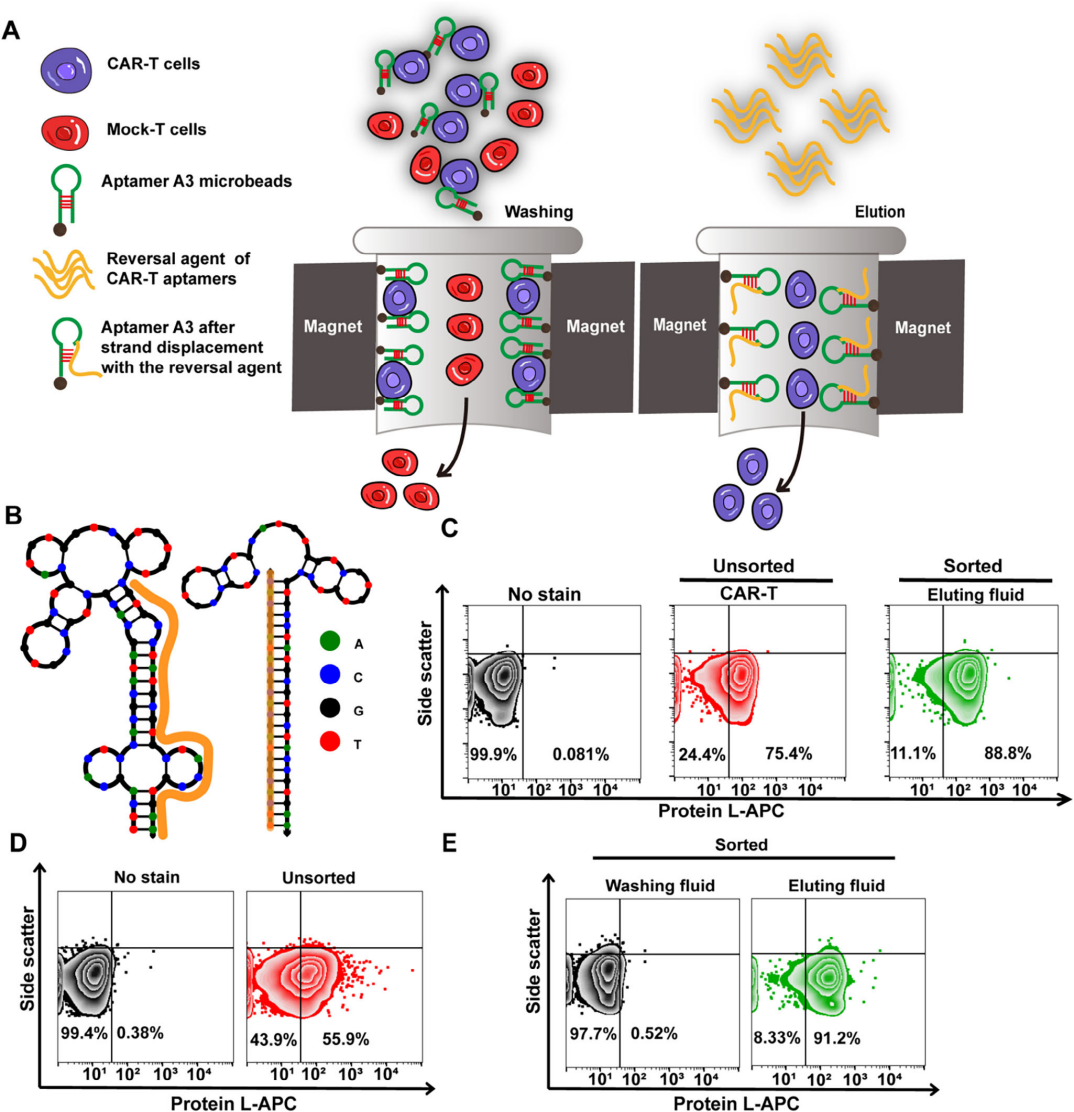
Traceless sorting of CAR‐T cells utilizing an Aptamer A3 ‐based approach. A) Schematic representation of the traceless sorting method for CAR‐T cells using Aptamer A3. Biotinylated Aptamer A3 preloaded onto Streptavidin‐magnetic beads to form Aptamer A3 microbeads. B) Predicted minimum free energy (MFE) secondary structure of the modified Aptamer A3, as determined by NUPACK. The orange line indicates the 23‐nt region that a complementary reversal agent (RA) was designed to anneal. Flow cytometry assay to detect C) the CAR‐positive rate of the CAR‐T cells before and after Aptamer A3 sorting, D) the expression rate of CAR in mixed cell populations before Aptamer A3 sorting, and E) the expression rate of CAR in mixed cell populations after Aptamer A3 sorting.

Aptamer A3 was highly selective and had no effect on the therapeutic function of CAR‐T cells, which made it possess promising potential in enriching CAR‐T cells and improving the positive rate of CAR. Aptamer‐based strategy for traceless cell isolation was preferred for cell separation process.^[^ ^26^ ^]^ The traceless CAR‐T cell enrichment strategy based on Aptamer A3 was shown in **Figure** **5**A. After co‐incubation with the Aptamer A3 microbeads, CAR‐T cells were captured by the Aptamer A3 microbeads and gathered around the magnetic frame. To release Aptamer A3 microbeads from CAR‐T cells, the designed reversal agent partly complemented to Aptamer A3 was introduced (Table S3, Supporting Information). NUPACK simulations revealed structural changes in the Aptamer A3 sequence before and after the use of complementary reversal agent, suggesting a potential for the competitive binding between the reversal agent and Aptamer A3 (Figure 5B). Corresponding reversal agent were employed for the traceless isolation of CAR‐T cells within an entirely synthetic framework with immobilized aptamers for the initial capture of CAR‐T cells, and the reversal agent for their release from CAR‐T cells. With aptamer enrichment, the positive rate of CAR‐T cells in eluting fluid was 88.8%, higher than that without aptamer sorting (75.4%) (Figure 5C). More importantly, we evaluated its performance on the CAR‐T cell enrichment in mixed cell sample. CAR‐T cells were mixed with Mock‐T cells (1:1) and the positive rate of CAR in the mixed cell population was measured to be 55.9% without aptamer enrichment (Figure 5D). Then, CAR‐T cells in mixed cell populations were enriched by the A3 aptamer microbeads and the CAR positive rate in eluting fluid was 91.2% while the CAR positive rate in washing fluid was only 0.52% (Figure 5E). Microscopic observation photographs in Figure S15 (Supporting Information) showed the cell clusters appeared around the magnetic beads after aptamer microbeads addition and disappeared after reversal agent introduction, indicating the capturing and releasing ability of Aptamer A3 and its complementary reversal agent to CAR‐T cells, demonstrating that Aptamer A3 facilitates efficient and specific traceless enrichment of CAR‐T cells in vitro.

Our previous study developed bispecific BC19 CAR‐T cells^[^ ^19^ ^]^ that target both BCMA and CD19, and initially assess their safety and effectiveness in patients with relapsed or refractory multiple myeloma. However, in that assay, peripheral blood was only monitoring, which provides limited information, leaving the viability and distribution of CAR‐T cells in major organs, lymphoid tissues, and bone marrow largely unexplored. CAR‐T cell therapy exhibits varied responses and unpredictable relapse rates. Monitoring of CAR‐T cell therapies in vivo can provide essential temporal and spatial data on CAR‐T cell populations, which remains a focal point for extensive investigation and ongoing research efforts. Several researchers have explored the utilization of reporter genes and ex vivo labeling techniques to enhance the monitoring of CAR T cell therapies.^[^ ^10^ ^]^ Despite the availability of various reporter gene‐based imaging techniques, all these methods require additional genetic engineering of CAR T cells to introduce a reporter gene, which may potentially result in on‐target off‐tumor toxicity.^[^ ^9a^ ^]^ While the ex vivo labeling approach circumvents genetic modification but is constrained by its inability to provide imaging at specific time points, thereby limiting its clinical applicability. The utilization of immune‐imaging offers several advantages over alternative approaches.^[^ ^14^ ^]^ First, it obviates the need for introducing a dedicated imaging reporter gene. Second, the administration of labeled “probes” at various time points post‐CAR T cell infusion enables repeated in vivo monitoring of CAR T cell activity.

A significant challenge in the in vivo immune‐imaging of CAR‐T cells is the identification of suitable target candidates to obtain the corresponding “probes”. The ideal target should exhibit selective expression within the CAR‐T cell population and have minimal expression in native cells. Moreover, the “probes” are expected to achieve non‐invasively monitoring of the biodistribution, expansion, and persistence of CAR‐T cells at tumor sites which can provide assistance to evaluate treatment success, potential failure, and off‐target toxicities. In this study, we reported the identification of a nanomolar affinity DNA aptamer, designated as Aptamer A3, which exhibits high affinity and specificity for CAR‐T cells. The proposed target of Aptamer A3 is the BCMA‐CD19‐His protein, which is exclusively expressed in CAR‐T cells and shows minimal expression in mock T cells. This Aptamer A3 was obtain through traditional Cell‐SELEX with slight modification.^[^ ^27^ ^]^ In our selection cycles, a negative selection step was added by introducing Mock‐T cells to reduce non‐specific binding sequences and the sequences that bind to common surface molecules shared by both CAR‐T cells and Mock‐T cells. After 11 cycles of screening, eight candidate aptamers were chosen and Aptamer A3 demonstrated high binding affinity with CAR‐T cells in the low nanomolar range. Considering the molecular difference between CAR‐T cells and Mock‐T cells, BCMA‐CD19‐His protein was testified a potential target for Aptamer A3. Different from the aptamer selection with single biomolecule as target, the Aptamer A3 generated by Cell‐SELEX in this study could bind CAR‐T cells in a real cellular environment, thereby enhancing its potential for in vivo immune‐imaging of CAR‐T cells.

Another essential factor to consider when designing novel immune‐imaging methods is the potential to disrupt CAR‐T cell functionality. Introducing reporter genes that encode foreign proteins poses a substantial risk of immunogenicity, which may restrict the therapeutic efficacy of CAR‐T cells. Additionally, incorporating additional genes into the construct could substantially interfere with CAR expression and/or functionality. Antibodies or antigens used as probes may induce biological effects upon binding to their targets, potentially disrupting the homeostasis and function of CAR‐T cells. Aptamers, which are well‐defined, exhibit low variability, can be easily modified the signal molecules, and possess long‐term storage stability, represent promising tools for the tracking of infused CAR‐T cells. Our reported Aptamer A3 had no effect on the CAR expression rate in CAR‐T cells and did not adversely impact the tumor‐killing ability of CAR‐T cells in vitro. These findings suggest the safety of our approach, which is consistent with previous studies. consistent with previous studies.^[^ ^28^ ^]^

It is necessary to test the serum stability of the aptamer before its application in vivo owing to flexible conformation of aptamers that may impede broad in vivo use of aptamers.^[^ ^29^ ^]^ The relatively good serum stability further strengthens Aptamer A3 use in vivo immune‐imaging of CAR‐T cells (Figure 4). In vivo fluorescence imaging with the Cy5‐labeled Aptamer A3 in xenograft tumor mouse model demonstrated that specific signal was observed at tumor region in the mouse model with CAR‐T cells injection while no significant fluorescence in the mouse model without CAR‐T cells. Furthermore, the fluorescence signal from Aptamer A3 in tumor region was co‐localized with CD8 antibody, overexpressed on CAR‐T cells, confirming the feasibility of tracking CAR T cells by Aptamer A3 in vivo. More importantly, CAR‐T cells in tumor region could be serially imaged for a month through repeated injection of Aptamer A3. Desirable monitoring of CAR‐T cell therapies should encompass the capability to track CAR‐T cell migration, interaction with antigen‐presenting tumor cells, and evaluate CAR‐T cell expansion and persistence at the tumor site.^[^ ^7^ ^]^ These results demonstrated Aptamer A3 has the potential to be an ideal probe for tracking CAR‐T cells in vivo. Indeed, aptamers such as sgc8 and AS1411 have been utilized for live cell tracking and have demonstrated progress in in situ tumor imaging in vivo and in the preclinical treatment of diseases.^[^ ^15^, ^30^ ^]^

It is important to distinguish between the self‐distribution and target‐specific distribution of nucleic acid aptamers for in vivo imaging. Tan's group reported a study on the human metabolic distribution of aptamers, which dynamically characterized the distribution and metabolism of aptamers in the human body using panoramic dynamic PET scanning and established a corresponding pharmacokinetic mode.^[^ ^18^ ^]^ The study developed a differential pharmacokinetic model by comparing imaging discrepancies between patients and healthy individuals or between diseased and normal tissues. In our study, to distinguish the in vivo self‐distribution of Aptamer A3 from its targeted distribution to CAR‐T cells, and to verify the in vivo fluorescence tracking capability of Aptamer A3 for CAR‐T cells in the Nalm6 tumor‐bearing mouse model, we established a control group (without CAR‐T cell injection) and an experimental group (with CAR‐T cell injection). Subsequently, Cy5‐labeled Aptamer A3 was injected at the same time point into both groups. The in vivo monitoring ability of Aptamer A3 for CAR‐T cells was evaluated by comparing small‐animal in vivo imaging results (Figure 4C) and ex vivo Cy5 fluorescence intensities in major organs (Figure 4D,E) between the control and experimental groups. Whole‐body PET imaging technology, through the use of specific probes, enables dynamic monitoring of the in vivo distribution and activity of CAR‐T cells. This allows for the evaluation of treatment responses, prediction of long‐term efficacy, and elucidation of mechanisms underlying treatment‐related toxicity, thereby providing critical data support for optimizing individualized cancer immunotherapy. In the future, we would plan to label the screened Aptamer A3 of CAR‐T cells radionuclides to achieve dynamic monitoring of the in vivo distribution and activity of CAR‐T cells.^[^ ^18^, ^31^ ^]^

Aptamers for cell isolation have also emerged as a very promising opportunity in CAR‐T cells production. One method reported for clinical‐scale T cells isolation for CAR‐T cells manufacturing is immunomagnetic positive enrichment.^[^ ^32^ ^]^ Traditional methods utilized antibodies as probes to form immunomagnetic beads for the separation of labeled CAR‐T cells and unwanted cells. Although this approach can achieve a high yield of CAR‐T cells, the nearly irreversible antibody labeling impairs T cell function, thereby reducing the efficacy of CAR‐T cells therapy.^[^ ^33^ ^]^ The aptamers share the highly selective nature of antibodies and can achieve traceless cell isolation by denaturing the secondary structure through complementary oligonucleotide binding.^[^ ^34^ ^]^ As expected, Aptamer A3 performed well in CAR‐T cells enrichment. The CAR expression rate was changed from 75.4% to 88.8% using aptamer‐based strategy for traceless cell isolation. Particularly, with 55.9% CAR expression rate in mixed cell population (CAR‐T cells: Mock‐T cells = 1:1) could raise to 91.2% after Aptamer A3 sorting, showing the promising potentials of Aptamer A3 e for purification of CAR‐T cells and large‐scale manufacturing of CAR‐T cells which is crucial to improve CAR‐T cells therapeutic efficacy.

## 3 Conclusion

In conclusion, we reported the discovery of a nanomolar affinity DNA aptamer named Aptamer A3, which showed affinity and specificity for CAR‐T cells. The potential target of Aptamer A3 is the BCMA‐CD19‐His protein. Our reported Aptamer A3 had no effect on the CAR expression rate in BC19 CAR‐T cells and did not adversely impact the tumor‐killing ability of CAR‐T cells, suggesting the safety of our approach. In vivo fluorescence imaging with Cy5‐labeled Aptamer A3 in xenograft tumor models showed specific signals in CAR‐T cell‐injected mice, but not in controls. For the first time, we demonstrated the feasibility of using an Aptamer‐based approach to enable in vivo imaging and tracking of CAR‐T cell migration in tumor‐bearing mice. BC19 CAR‐T cells were monitored for a month through direct labeling strategies, which demonstrated Aptamer A3 had the potential for long‐term continuous monitoring of CAR‐T cells. In addition, a CAR‐T cells enrichment platform was constructed using Aptamers A3, which effectively increased the positive rate of CAR‐T cells. Our results indicated that the Aptamer has the potential to serve as a highly specific and effective probe for in vivo CAR‐T cell tracking. In the future, this SELEX technology could be employed to identify a broader range of aptamers for various types of CAR‐T cells, thereby enhancing the feasibility and precision of imaging studies focused on these CAR‐T cells. Building on this strategy, the screened CAR‐T cells aptamers labeled with PET probes would have broad applications for tracking CAR‐T cells in patients, providing insights into treatment success, potential failure, and off‐target toxicities.

## Conflict of Interest

The authors declare no conflict of interest.

## Author Contributions

M.C. and P.C. contributed equally to this work. M.C. and P.C. performed conceptualization, investigation, performed the experiments, formal analysis, and wrote the original draft. Z.Z. performed visualization and assisted with some experiments. R.H. and D.L. performed some of the experiments. M.S. provided advice and reviewed the manuscript. J.L. performed data curation, investigation, wrote, reviewed, and edited the final manuscript. K.X. acquired funding acquisition and wrote, reviewed, and edited the final manuscript. J.Z. acquired funding acquisition and performed supervision.

## Supporting information



Supporting Information

## Data Availability

The data that support the findings of this study are available in the supplementary material of this article.
