# Supplementary material for: Development of a DNA Aptamer‐Based Approach to Noninvasively Image CAR‐T Cells In Vivo and Traceless Enrichment In Vitro
Source: Adv Sci (Weinh). 2025 May 11;12(28):2506746. doi: 10.1002/advs.202506746 (PMC12302554; doi:10.1002/advs.202506746)
Supplement: Supplementary file 1 — Supporting Information [file ADVS-12-2506746-s001.docx]

**Development of a DNA Aptamer-Based Approach to Noninvasively Image CAR-T Cells in Vivo** **and Traceless Enrichment in Vitro**

Minghui Chen^1,3,#^, Pengzhao Chang^1,3,#^, Zhen Zhang^1,3^, Dan Liu^2,4,5^, Rui Hou^2,4,5^, Ming Shi^2,4,5,*^, Jingjing Li^1,3,*^, Kai Xu^1,3,*^, Junnian Zheng^2,4,5,*^

1. School of Medical Imaging, Xuzhou Medical University, Xuzhou, Jiangsu 221004, China.

2. Cancer Institute, Xuzhou Medical University, China.

3. Department of Radiology, Affiliated Hospital of Xuzhou Medical University, Xuzhou 221006, China.

4. Center of Clinical Oncology, The Affiliated Hospital of Xuzhou Medical University, China.

5. Jiangsu Center for the Collaboration and Innovation of Cancer Biotherapy, Xuzhou Medical University, China.

^#^ M. Chen and P Chang contributed equally to this work.

* *E-mail addresses:* mingshi@xzhmu.edu.cn (Ming Shi), qingchao0124@163.com (Jingjing Li), xkpaper@163.com (Kai Xu), jnzheng@xzhmu.edu.cn (Junnian Zheng)

**CONTENTS**

**Materials and Methods**…………….……………………………………….……....….S3

**PCR and gel electrophoresis results of Cell-SELEX procedures** **(Fig. S1)** ………...S8

**Flow cytometry analysis of the binding efficiency of aptamers (Fig. S2)** ………......S9

**Sequential predicted motif of the 11th round aptamer pool sequences (Fig. S3)** …...S10

**Flow cytometry analysis of the binding specificity of the aptamers (Fig. S4)** ...........S11

**Flow cytometry binding curve of the selected aptamers (Fig. S5)** ………………......S12

**CMI of CAR-T cells by the selected aptamers (Fig. S6)** …………………………….S13

**CMI of CAR-T cells by the Cy5-labeled Aptamer A3 (Fig. S7)** ……………………S14

**SPR results of Aptamer A3 for protein affinity (Fig. S8)** ………….…………….…S15

**The effects of different concentrations aptamer A3 on CAR expression rates and activation marker profiles in CAR-T cells (Fig. S9)** ………….………….…………S16

**Impact of Aptamer A3 on the *in viv*o killing efficacy of CAR-T cells (Fig. S10)**.…S17

**Full-size image of gel for Figure 3E (Fig. S11)**…………….……………………….…S18

**FL images of mice injection of random library after CAR T cells (Fig. S12)** …….....S19

**Full-size images of gel for Figure 4A-B (Fig. S13)**…………….…………….………S20

**FL images of major organs of mice injection of A3 on day 14 (Fig. S14)** ……..……..S21

**Microscopic observation of CAR-T cell binding to the MNBs (Fig. S15)** ……...…...S22

**Experimental conditions in rounds of CAR-T cell SELEX (Table. S1)** ……...........S23

**Detailed sequences of the selected aptamer candidates (Table. S2)** ………………..S24

**Sequences of aptamers and reversal agent in experiments (Table. S3)** ……...........S25

**Materials**

Lonza X-VIVO 15 medium (Lonza, Basel, Switzerland) and fetal bovine serum (FBS; Hyclone, USA) were purchased from Shanghai Universal Biotech Co., Ltd (Shanghai, China). Recombinant Human IL-2, IL-7 and IL-15 were purchased from Thermo Fisher Scientific (Massachusetts, USA). Biotinylated Recombinant Protein L, His, AvitagTM was provided from ACROBiosystems (Beijing, China). 4 % paraformaldehyde, phosphate buffer solution (PBS), 4', 6-diamidino-2-phenylindole (DAPI) solution (>90 %), streptavidin magnetic beads and streptavidin agarose were purchased from Beyotime Institute of Biotechnology (Shanghai, China). Cell counting plate was purchased from Thermo Fisher Scientific (Massachusetts, USA). MgCl_2_.6H_2_O and D-PBS were provided by MeilunBio Biotechnology Co., Ltd (Dalian, China). APC-Streptavidin was purchased from BioLegend (California, USA). APC-Streptavidin was purchased from BioLegend (California, USA). BCMA-CD19-His protein was purchased from Sino Biological co., Ltd (Beijing China). APC anti-human CD3 antibody (OKT-3), FITC anti-human CD4 antibody (SK3), PE/Cyanine7 anti-human CD8a antibody (OKT-8), PE anti-human CD25 antibody (BC96) and PE anti-human CD69 antibody (FN50) were purchased from Elabscience Biotechnology Co., Ltd. (Wuhan China). Cell-SELEX single chain preparation kit and micro nucleic acid dialysis membrane were purchased from Aptamy Biotechnology co., Ltd (Hefei, China). Glucose and n-butyl alcohol were provided from Sinopharm Chemical Reagent Co., Ltd (Shanghai China). 2×SYBR Green qPCR Mix was purchased from Toyobo life science Co., Ltd. Oligonucleotides used in this study were commercially supplied by Sangon Biotech (Shanghai, China). The initial randomized library and PCR primer sequences were designed as follows:

Randomized library:5'-TTCAGCACTCCACGCATAGC-36N-CCTATGCGTGCTACCGTGAA-3',

where 36N represents the 36 randomized nucleotides.

Forward primer: 5'-FAM-TTCAGCACTCCACGCATAGC-3'.

Reverse primer:5'-AAAAAAAAAAAAAAAAAAA-Spacer18-TTCACGGTAGCACGCATAGG-3'.

Buffer solution: Washing buffer: 4.5 g of glucose and 5 mL of 1 M MgCl_2_ were added to 1 L of D-PBS. Binding buffer: 4.5 g of glucose, 100 mg tRNA, 1 g BSA and 5 mL of 1 M MgCl_2_ were dilluted in 1 L of D-PBS. The obtained buffers were stored at 4 ℃ for up to 1 month.

Software: (i) FlowJo (analysis of flow cytometry data). (ii) DNAMAN, MEME (multiple sequence alignment and phylogenetic tree). (iii) NUPACK (aptamer structure prediction). (iv) SigmaPlot for apparent dissociation constant determination.

**Cell culture and animal models.** Nalm6 cells and U266 cells were purchased from Wuhan Pricella Biotechnology Co., Ltd (Wuhan, China). CAR-T cells and Mock-T cells were cultured in LONZA X-VIVO 15 medium containing 200 U/mL IL-2, 10 ng/mL IL-7, 10 ng/mL IL-15 and 10 % heat-inactivated FBS. Nalm6 cells, Jurkat cells and U266 cells were cultured in RPMI 1640 medium containing 10 % heat-inactivated FBS. RAW264.7 cells, MAEC cells and GL-261 cells were cultured in DMEM medium containing 10 % FBS and all cells were maintained in a humidified incubator with 5 % CO_2_ at 37 °C. The severely immunodeficient female mice (NOG) were purchased from Beijing Vital River Laboratory Animal Technology Co, Ltd (Beijing, China). 2 × 10^6^ Nalm6 cells/mouse were subcutaneously to the right lateral thigh to establish Nalm6 tumor-bearing mice models.

**Cell-SELEX procedures.** The initial ssDNA library was dissolved with phosphate buffered saline. Before each incubation, the library was heated at 95 °C and cooled on ice for DNA denaturation. After incubation with CAR-T cells for 1 h at 4 °C, washing was performed to remove unbound sequences and harvest the ssDNA binding cells. DNA fragments were separated from the cell-DNA complexes through denaturation at 95 °C for 10 min. The sequences were isolated from cell precipitation by low temperature centrifugation and then used for PCR amplification. The PCR amplification procedure was as follows: 10-15 cycles of (95 °C for 30 s, 60 °C for 30 s, and 72 °C for 30 s), followed by 3 min at 72 °C. Since the reverse primers we used had lengthened nucleic acid fragments, the short and long chain separation method was used to prepare the single strand. The single strand library was concentrated with n-butanol and then desalted by micro nucleic acid dialysis membrane so they were ready for the next round of screening. After positive selection in evolutionary library, the eluted sequences were incubated on ice with Mock-T cells for 1 h and the liquid supernatant containing ssDNA with affinity to CAR-T cells sequences were selected for PCR amplification. Conditions gradually became more stringent from the first to 11th rounds to enhance the screening efficiency by decreasing the reaction time with CAR-T cells (from 1 h to 20 min) and CAR-T cells number (from 5 × 10^6^ to 0.5 × 10^6^ cells), while increasing the incubation time with Mock-T cells (from 30 min to 2 h), the number of Mock-T cells (from 1 × 10^6^ to 2 × 10^6^ cells), and the number of washing (from one to four) (Table S1). The final ssDNA library of the 11th round was directly sequenced by Sangon Biotech Co., Ltd (Shanghai, China).

**Flow Cytometric analysis.** In order to enrichment analysis, CAR-T cells and Mock-T cells were separately incubated with each round of the selected sequences (250 nmol/L final concentration) in binding buffer at 4 °C in the dark for 40 min. The initial library was used as the negative control. The cell suspension was centrifuged and washed twice to remove unbound sequences. Samples containing cells and selected sequences were evaluated using flow cytometry (Guava easyCyte, USA). This above binding experiment was carried out three times independently.

**Specificity of aptamers.** To investigate the specific recognition of candidate aptamers for other cell lines, we subjected several different cell lines (Nalm6, U266, Jurkat, Raw264.7, MAEC, GL-261) to flow cytometry analysis, the final concentration of the candidate aptamers was 250 nmol/L. Moreover, the specific recognition of Aptamer A3 at different concentrations (500 nmol/L, 1000 nmol/L, 2000 nmol/L) against six different cell types was investigated. Cells were treated with initial library tagged with FAM served as a control for nonspecific adsorption.

**Affinity of aptamers.** To measure the dissociation constant (K_D_) of the candidate aptamers, a series of concentration gradients of FAM-labeled aptamers (1 nmol/L, 2 nmol/L, 5 nmol/L, 10 nmol/L, 20 nmol/L, 50 nmol/L, 100 nmol/L, 200 nmol/L final concentration) were incubated with the target cells on ice for 40 min. Washing buffer was used to wash the cells three times. After resuspending in binding buffer, CAR-T cells were analyzed by flow cytometry. The relative fluorescence intensity at each concentration was recorded. Random libraries were used as controls. SigmaPlot software (Jandel Scientific, UK) was utilized for data plotting and curve fitting to estimate the K_D_ value, through the equation Y = B_max_X / (K_D_ + X), where X, Y, and Bmax were the aptamer concentration (nmol/L), fluorescence intensity at each concentration, and the maximum fluorescence intensity, respectively.

**Effect of temperature on aptamers binding ability.** To determine if temperature shift would change the affinity of aptamer, we prepared CAR-T cells in washing buffer and incubated them with aptamers at 4 °C or 37 °C for 40 min, respectively. The final concentration of FAM-labeled aptamers was 250 nmol/L and the initial library served as the control group. All the stained CAR-T cells were subjected to flow cytometry analysis.

**Confocal microscopy imaging of cells.** To achieve the *in vitro* tracking of CAR-T cells, CAR-T cells were incubated with 250 nmol/L FAM-labeled candidate aptamers for 40 min at 4 °C or 37 °C. After washing with PBS, CAR-T cells were fixed with 4 % formaldehyde-glutaraldehyde for 10 min at room temperature, stained with DAPI and imaged using a confocal laser microscope (CLSM) (Leica Stellaris 5, Germany). Mock-T cells served as a control group.

**Identification of aptamer target.** After washing the CAR-T cells twice with PBS, the cells were treated with trypsin for 5 min. Complete medium was then added to cell suspension to stop trypsin action. The liquid supernatant was separated by centrifugation (1200 rpm, 5 min). The cell sediment was added with 200 μL Aptamer A3 (250 nmol/L) and incubated for 40 min. The mixture of CAR-T cells and Aptamer A3 were washed twice with washing buffer and then detected by flow cytometry.

RIPA lysis buffer was mixed with PMSF at a 100 : 1 to prepare cell dissociation buffer. CAR-T cells were washed twice with PBS and then centrifuged. Cell dissociation buffer was mixed with CAR T cells and incubated on ice for 20 min. The protein solution was obtained by centrifugation (12000 rpm for 10 min), which was then incubated with biotin-labeled Aptamer A3 or random library (250 nmol/L final concentration) for 40 min. After centrifugation, streptavidin agarose was added and incubated at 37 ℃ for 1 h and washed twice with washing buffer. Subsequently, the aptamer A3-binding protein was eluted through high-temperature denaturation (following centrifugation after heating) and analyzed via SDS-PAGE to verify the A3 aptamer targeting protein.

The affinity between the Aptamer A3 and BCMA-CD19-His protein was analyzed by surface plasmon resonance (SPR). The SPR studies were carried out on a Biacore 1K instrument (Cytiva, Uppsala, Sweden). BCMA-CD19-His protein was coupled to the CM5 chip at a concentration of 20 μg/mL via sodium acetate (pH 5.0). The concentrations of Aptamer A3 were established at 7.8125, 15.625, 31.25, 62.5, 125 nmol/L to assess its affinity with BCMA-CD19 protein using multi-cycle kinetics. The obtained data were processed with BIAevaluate software (version 2.0.2 Plus Package) from Cytiva (Uppsala, Sweden). The equilibrium dissociation constant (K_D_, M) was determined by fitting the experimental data with the 1:1 binding model.

**Functional validation of CAR-T cells-Aptamer A3.** Verification of CAR expression on CAR-T cells-Aptamer A3. CAR-T cells were incubated with 250 nmol/L Aptamer A3 for 40 min to form CAR-T cells- Aptamer A3. Then, CAR-T cells-Aptamer A3 were incubated with anti-protein L (ACRO Biosystems) human primary antibody in PBS solutions containing 2 % bovine serum albumin (BSA) at 4 ℃ for 30 min. Subsequently, cells were washed, resuspended in PBS, reacted with anti-APC Streptavidin (Biolegend) secondary antibody for 30 min in dark, and subjected to flow cytometry. Mock-T cells served as a control group. The CAR expression rate of CAR-T cells without aptamers incubation was detected with the same procedure as above.

**Assessment of CAR-T cells-aptamers activation.** In order to confirm the activation of CAR-T cells-Aptamer A3, U266 and Nalm6 cells were chosen as targets. U266 and Nalm6 cells were incubated with CAR-T cells or CAR-T cells-aptamers (CAR-T cells were incubated with aptamers A1, A2, A3, A4, A5, A6, A7, A8 at concentrations of 250 nmol/L, respectively), at an effector T cells to target cells (E:T) ratio of 5:1 for 24 h, respectively. Then, CAR-T and CAR-T cells-Aptamer A3 were harvested and stained with APC anti-human CD3 antibody, FITC anti-human CD4 antibody, PE/Cyanine7 anti-human CD8a antibody, PE anti-human CD25 antibody and PE anti-human CD69 antibody for 30 min at 4 ℃. Flow cytometric analysis was used to assess the expression of activation markers (CD25 and CD69) in CAR-T cells and CAR-T cells-Aptamer A3.

**In vitro cytotoxicity test.** U266 and Nalm6 cells were incubated with CAR-T or CAR-T cells-Aptamer A3 (CAR-T cells were incubated with Aptamer A3 at concentrations of 250 nmol/L and 2000 nmol/L, respectively) at E:T ratios of 1:1, 2:1, and 5:1 for 24 h, respectively. Then, the cells were stained with LDH kit for cytotoxicity evaluation and the absorbance at 490 nm was measured by the microplate reader (SpctraMax i3, Molecular Devices, USA).

**Tumor killing experiment of CAR-T cells incubated with Aptamer A3 in vivo**. 2 × 10^6^ Nalm6 cells were injected subcutaneously to the right lateral thigh of the severely immunodeficient female mice (NOG) to establish Nalm6 tumor-bearing mice models. The mice were randomly divided into 3 groups (n=4): PBS (control), CAR-T, CAR-T cells-Aptamer A3 (tail intravenous injection). After 4 days of tumor implantation, each mouse was injected 1 × 10^7^ CAR-T cells or CAR-T cells-Aptamer A3 through tail vein. Aptamer A3 (250 nmol/L) was incubated with CAR-T cells for 40 min to form CAR-T cells-Aptamer A3. The weight of mice and the tumor volume were measured every two days. The volume is calculated as V=d^2^ × L/2 (d: the width of tumor, L: the length of tumor). All mice were euthanized for tumor isolation on day 12.

**Stability of aptamers in serum.** DNA Aptamer A3 (2000 nmol/L final concentration) was incubated at 37 ℃ for 2 hours in various concentrations of human serum (0%, 20%, 50%, and 100%). Additionally, it was incubated at a 20% serum concentration for 2, 4, 8, 12, and 24 hours. Each sample was denatured at 95 ℃ and incubated for 5 min. The relative contents of aptamers after different treatments were detected by 3 % agarose gel. The images were showed using a gel imager (Bio-Rad USA) and analyzed by Image Lab (Bio-Rad USA).

**Tracking of CAR-T cells in Nalm6 tumor-bearing mice models.** Each tumor-bearing mouse was injected intravenously with 200 μL PBS containing 2 × 10^6^ CAR-T cells. After injection of CAR-T cells for 12 h, all mice were depilated and anesthetized with isoflurane. Then, 200 μL of PBS containing 4.5 nmol Cy5-labeled Aptamer A3 was injected into the tail vein as the experimental group, whereas an equal volume of Cy5-labeled Aptamer A3 was injected into the mice without CAR-T cells as the control group. The fluorescence signals in tumor-bearing mouse were monitored from 15 min to 140 min using the IVIS Lumina S5 Imaging System (PerkinElmer Health Sciences, MA, USA). Then, the tumor-bearing mice were euthanized, and heart, kidney, liver, spleen and tumor were dissected for in vitro fluorescence imaging. As for long-term monitoring of CAR-T cells in vivo, Nalm6 tumor-bearing mice models were injected with 2 × 10^6^ CAR-T cells on day 0. Cy5-labeled Aptamer A3 was injected into the mice with CAR-T cells on day 7, day 14, day 21, and day 28 through the tail vein. Each fluorescence signal acquisition was performed at 30 min post-injection of Cy5-labeled Aptamer A3 in the same way as above. Fluorescence images were analyzed by using Living Image Software (Perkin Elmer, MA, USA).

**Traceless enrichment of CAR-T cells. The CAR positive rate of CAR-T cells was first measured by flow cytometry.** Then, to show the potential of Aptamer A3 in traceless enrichment of CAR-T cells, the biotin-modified aptamers were incubated with streptavidin magnetic beads overnight at 4 ℃ and washed twice with PBS to obtain aptamer microbeads. CAR-T cells were incubated with aptamer microbeads at 37 ℃ for 30 min under mild rotation. Afterward, CAR-T cells population were washed with binding buffer and isolated using a magnetic frame. To separate aptamers from CAR-T cells, 10-fold reverse complementary chain agent was applied to cells labeled with aptamer microbeads and gently rotated at 37 ℃ for 1 h. The cell suspension was washed with PBS after passing through the magnetic frame and the eluting fluid containing the cell fractions was collected for assay of CAR positive rate.

The potential of Aptamer A3 in traceless of CAR-T cells in mixed cell populations was evaluated in this assay. The CAR positive rate of CAR-T cells in mixed cell populations (2 × 10^6^ mixed cells, CAR-T cells : Mock-T cells = 1 : 1) was first measured by flow cytometry. Then, mixed cells (CAR-T cells : Mock-T cells = 1 : 1) were incubated with aptamer microbeads at 37 °C for 30 min under mild rotation. Afterward, mixed cell population were washed with binding buffer and CAR-T cells in the mixed cell population were captured using a magnetic frame and the washing fluid was collected for assay of CAR positive rate. A 10-fold reverse complementary chain agent was applied to cells labeled with aptamer microbeads and gently rotated at 37 ℃ for 1 h. Then, the cells fractions were separated from aptamer microbeads and cells using an eluting fluid. Similarly, the eluting fluid containing the cell fractions was collected for assay of CAR positive rate.

**Ethical approval for the animal experiments.** All animal experiments were performed in compliance with the Principles of Laboratory Animal Care issued by the People’s Republic of China and the Guidelines for the Care and Use of Laboratory Animals established by Xuzhou Medical University, China (Permission No. 202406T003).


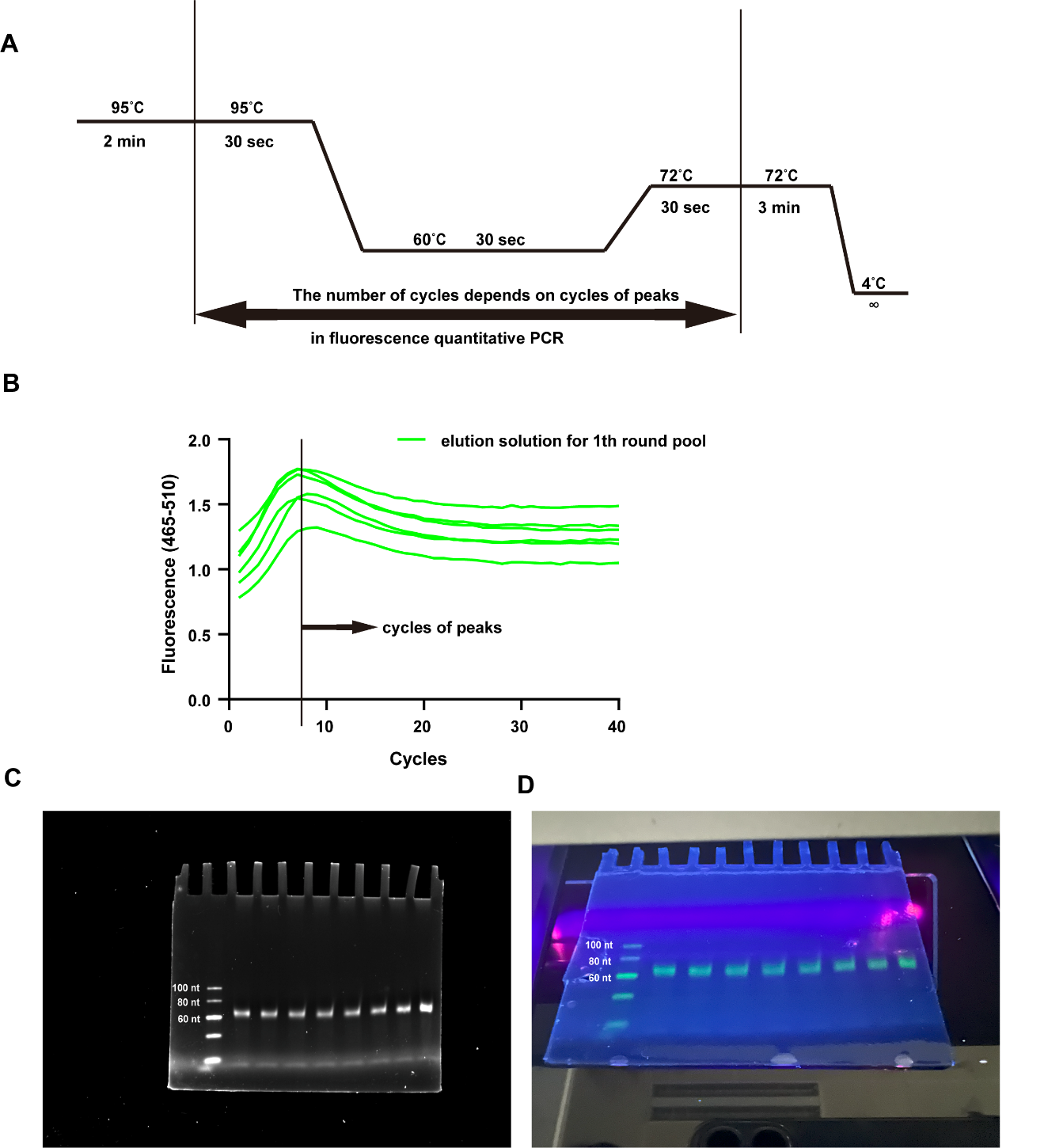


Figure S1. (A) Schematic illustration of cycle number optimization for PCR. (B) Amplification Curve of 1th round pool during six independent replicates Cell-SELEX experiments (elution: supernatant after negative selection, containing bound sequences with CAR-T cells). (C) Sodium dodecylsulphate polyacrylamide gel electrophoresis image of PCR products, (D) Sodium dodecylsulphate polyacrylamide gel electrophoresis image under the light of 254 nm ultraviolet lamp. Each well of the gel corresponds to identical products, representing ssDNA preparations derived from the PCR product of the 1th round library.


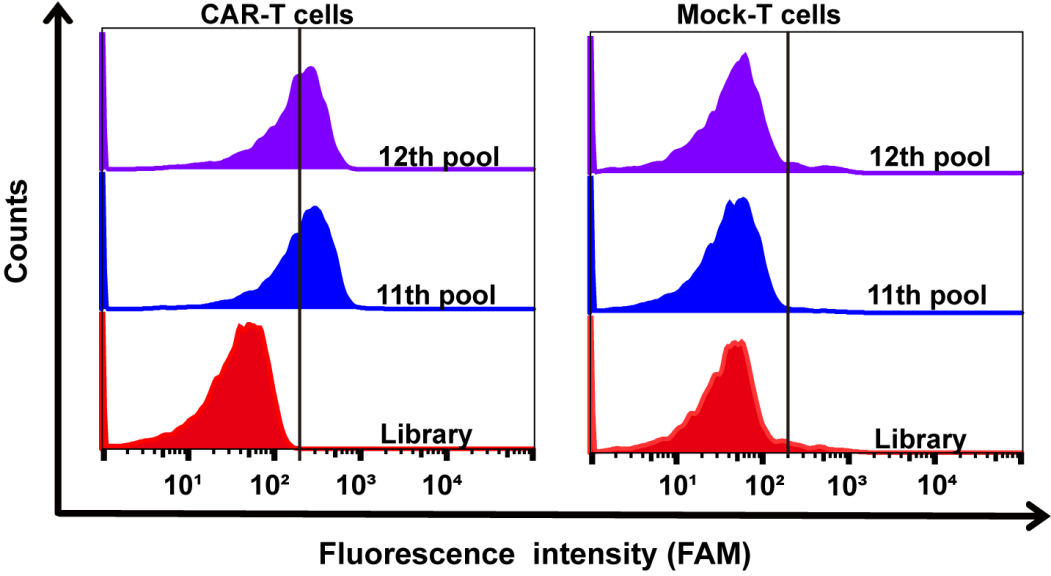


Figure S2. The binding performance of aptamers, ssDNA library, 11th pool and 12th pool to CAR-T cells and Mock-T cells by Flow Cytometry.


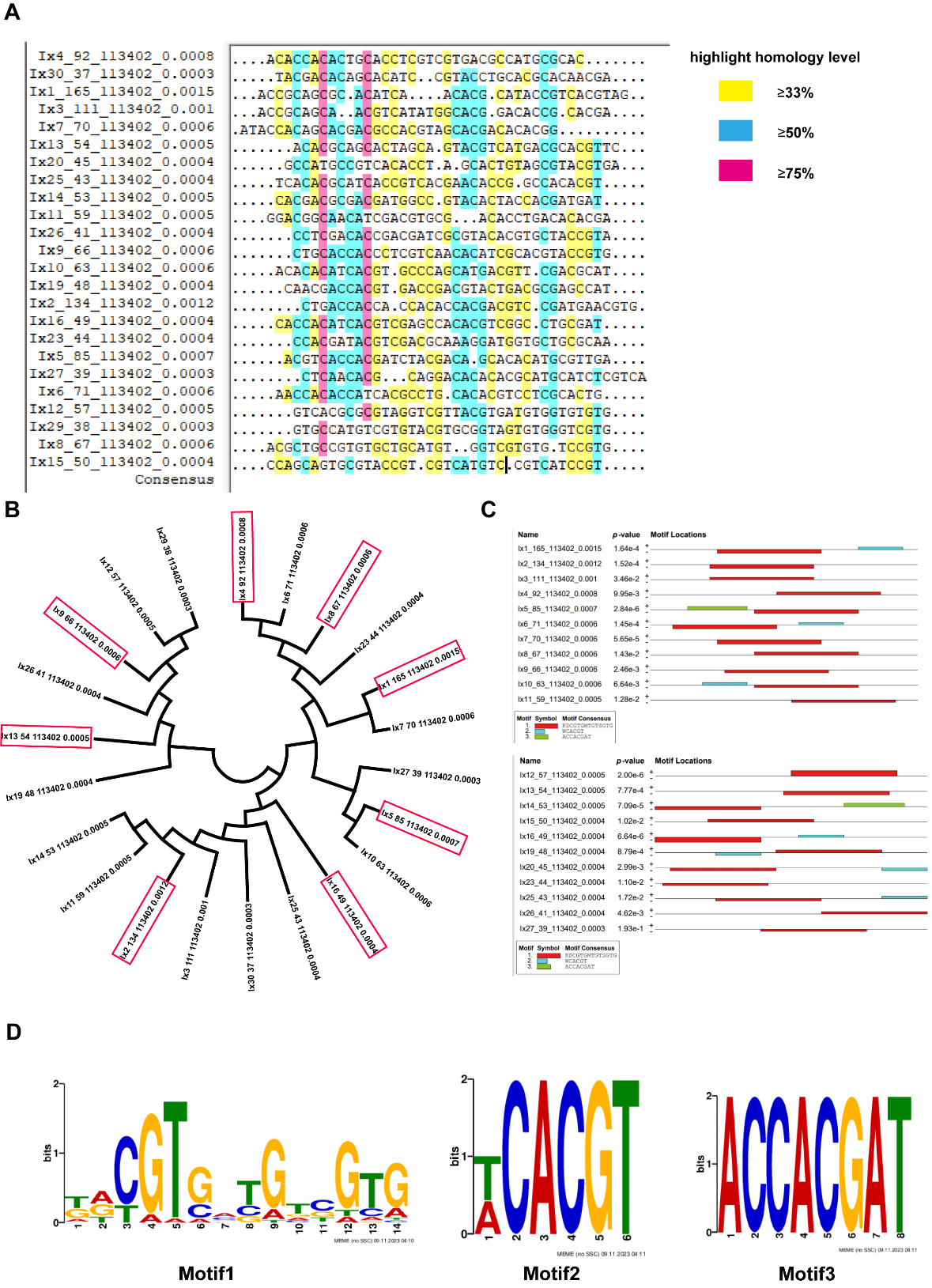


Figure S3. (A) DNA primary sequence alignment analysis of the top 30 sequences with the highest number of repeats in the sequencing results using DNAMAN. (B) The evolutionary tree of DNA sequence, where candidate sequences are in red boxes. (C) Binding motifs in 22 highly homologous sequences. (D) Binding motifs predicted using MEME analysis (MEME-suite.org).


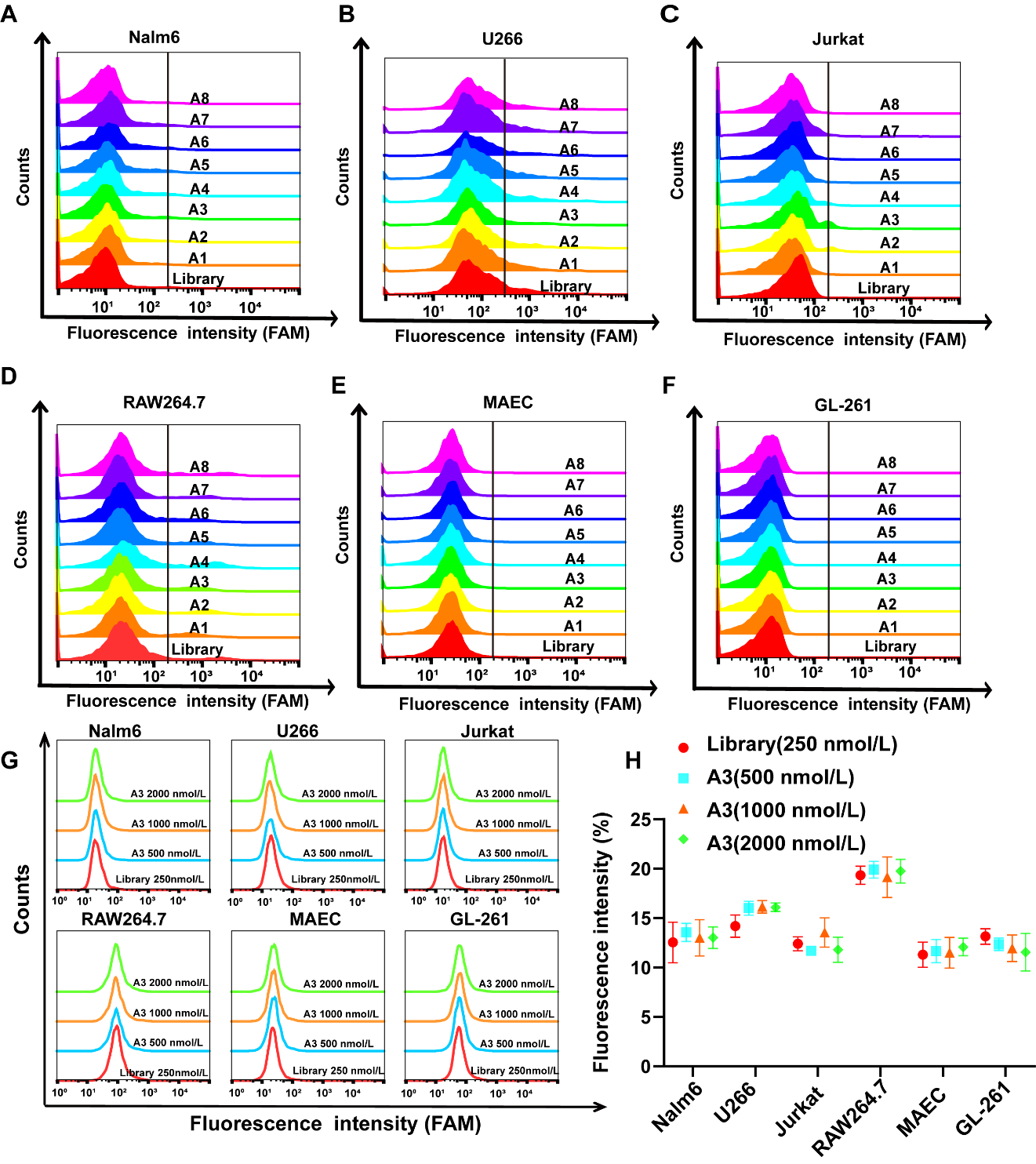


Figure S4. Binding specificity assay of the eight selected aptamer candidates (250 nmol/L) to different cells by Flow Cytometry (A) Nalm6 cells, (B) U266 cells, (C) Jurkat cells, (D) RAW264.7 cells, (E) MAEC cells, (F) GL-261 cells. (G) Flow cytometry was used to conduct a binding specificity assay of Aptamer A3 at different concentrations against six different cell types. (H) Quantitative analysis of Flow Cytometry fluorescence intensity for Aptamer A3 at different concentrations against six different cell types. Random library was used as a control group. Error bars represented the standard deviation (n=3).


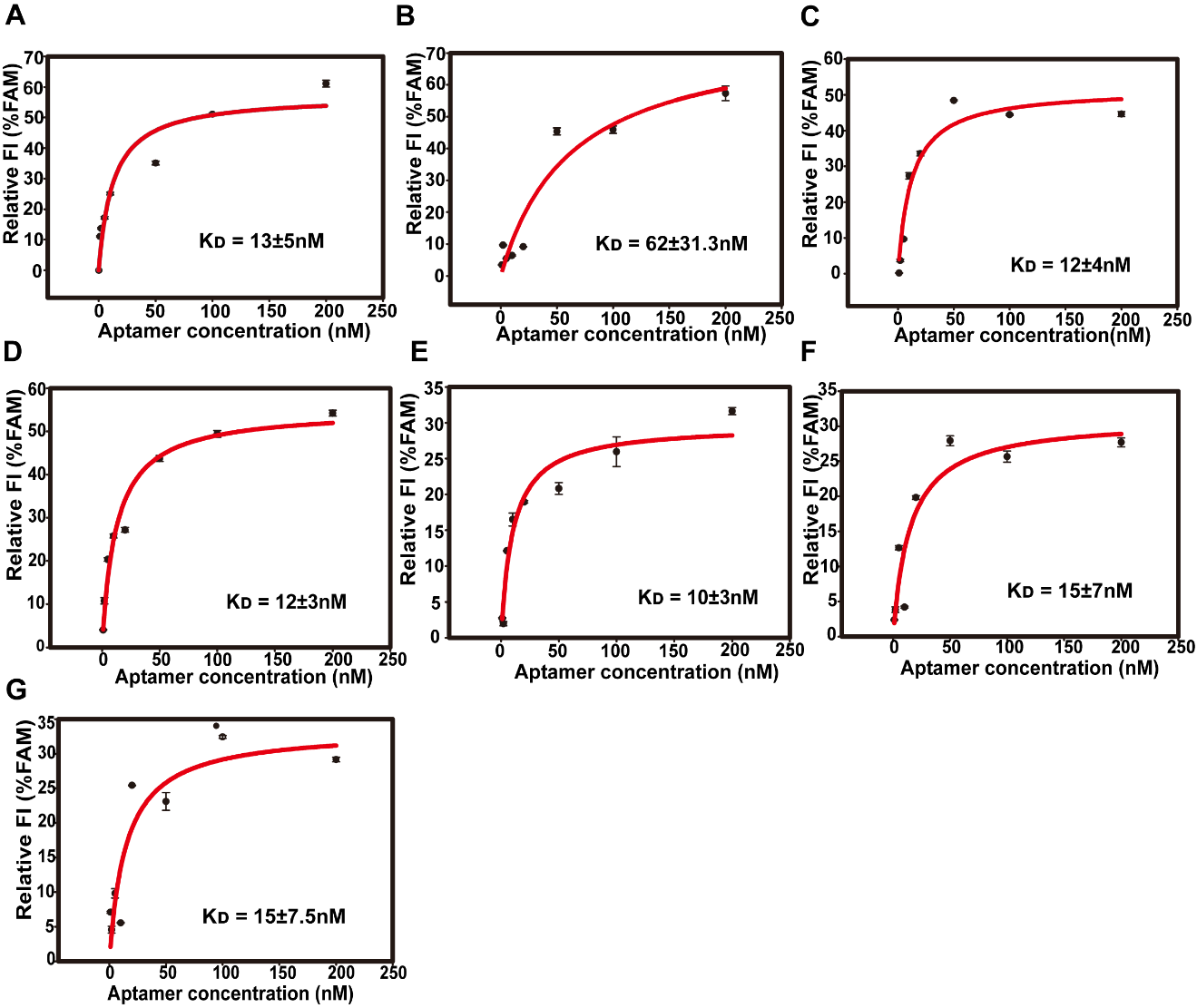


Figure S5. Flow cytometry binding curve of the eight selected aptamer candidates. (A) Aptamer A1, (B) Aptamer A2, (C) Aptamer A4, (D) Aptamer A5, (E) Aptamer A6, (F) Aptamer A7, (G) Aptamer A8. Error bars represented the standard deviation (n=3).


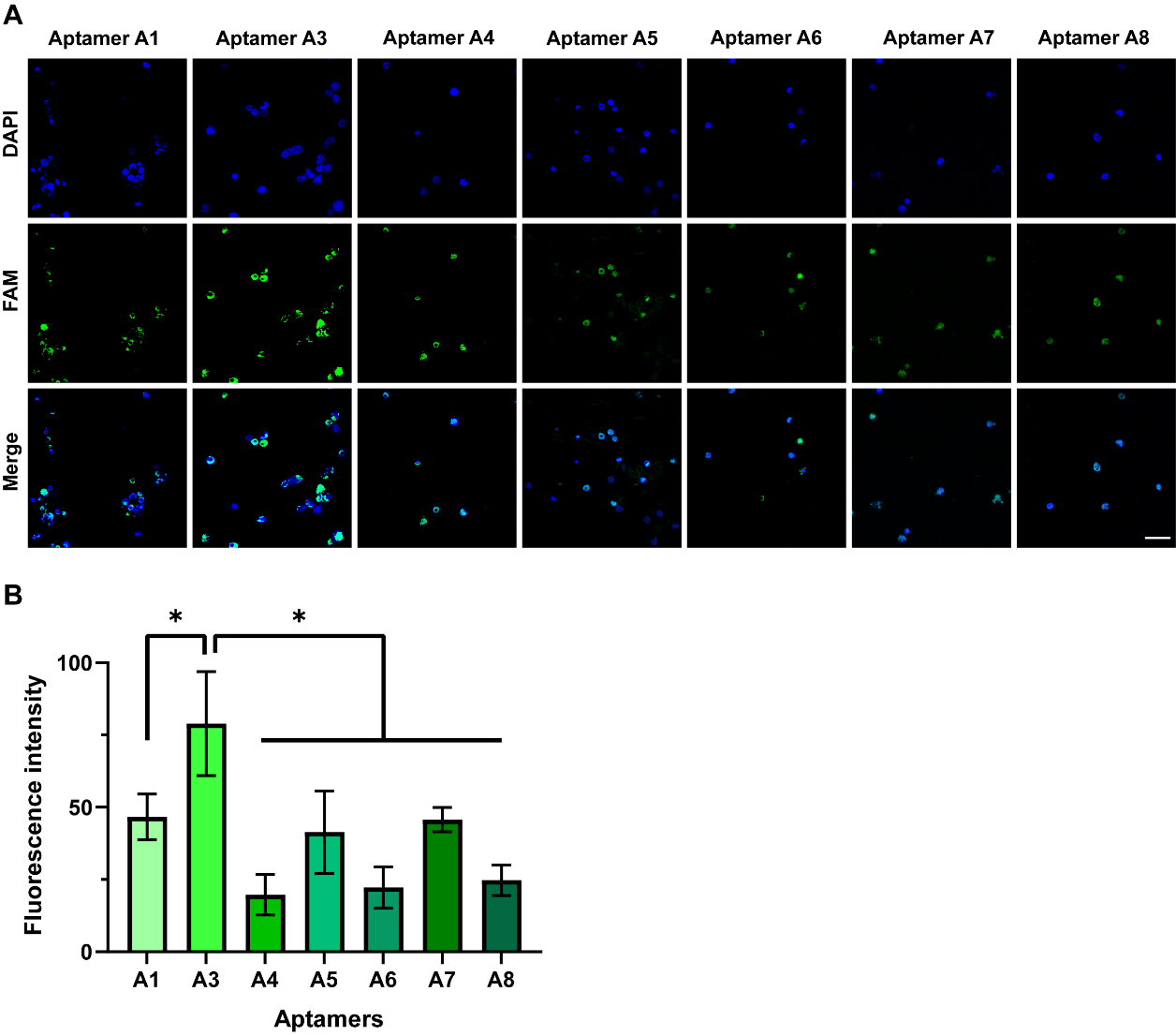


Figure S6. (A) Confocal microscopy imaging of CAR-T cells stained by the seven selected aptamer candidates (A1, A3, A4, A5, A6, A7, A8). Blue and green signals represent DAPI and FAM-labeled aptamers, respectively. Scale bar: 20 μm. (B) Quantitative analysis of average fluorescence intensities in Confocal images of CAR-T cells after incubation with the seven selected aptamer candidates. Error bars represented the standard deviation (n=3). *P < 0.05.


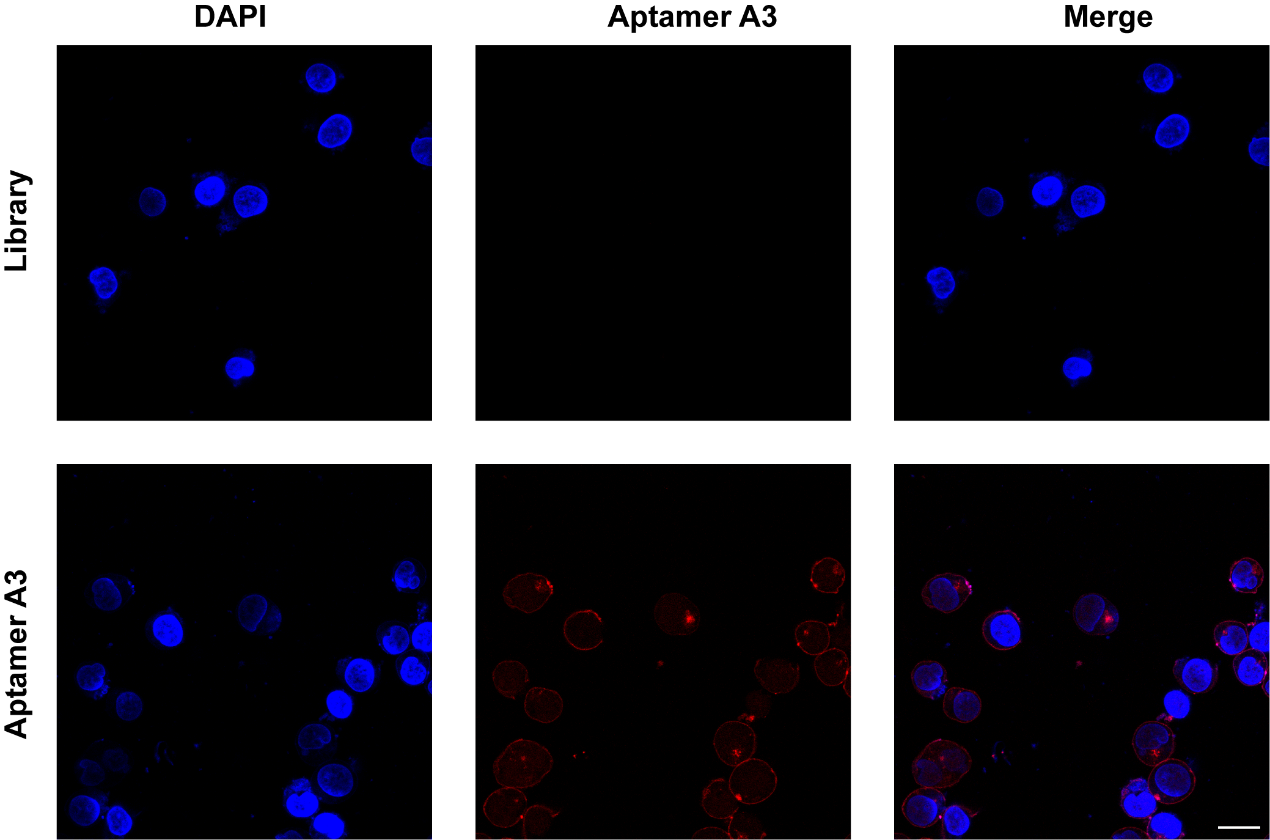


Figure S7. Confocal images of CAR-T cells stained with DAPI (blue) and co-cultured with Aptamer A3 (red). Blue and red signals indicated DAPI and Cy5-labeled Aptamer A3, respectively. Scale bar: 20 μm..


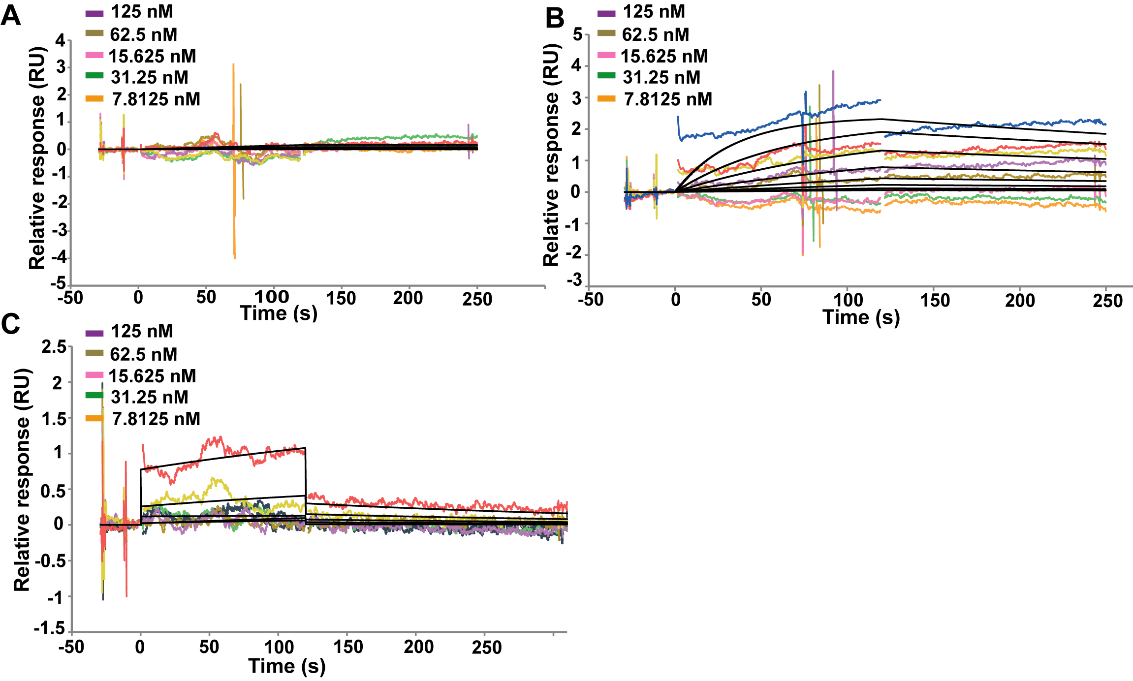


Figure S8. SPR results of Aptamer A3 for (A) His-tag protein, (B) BCMA CAR protein, and CD19 CAR protein affinity.


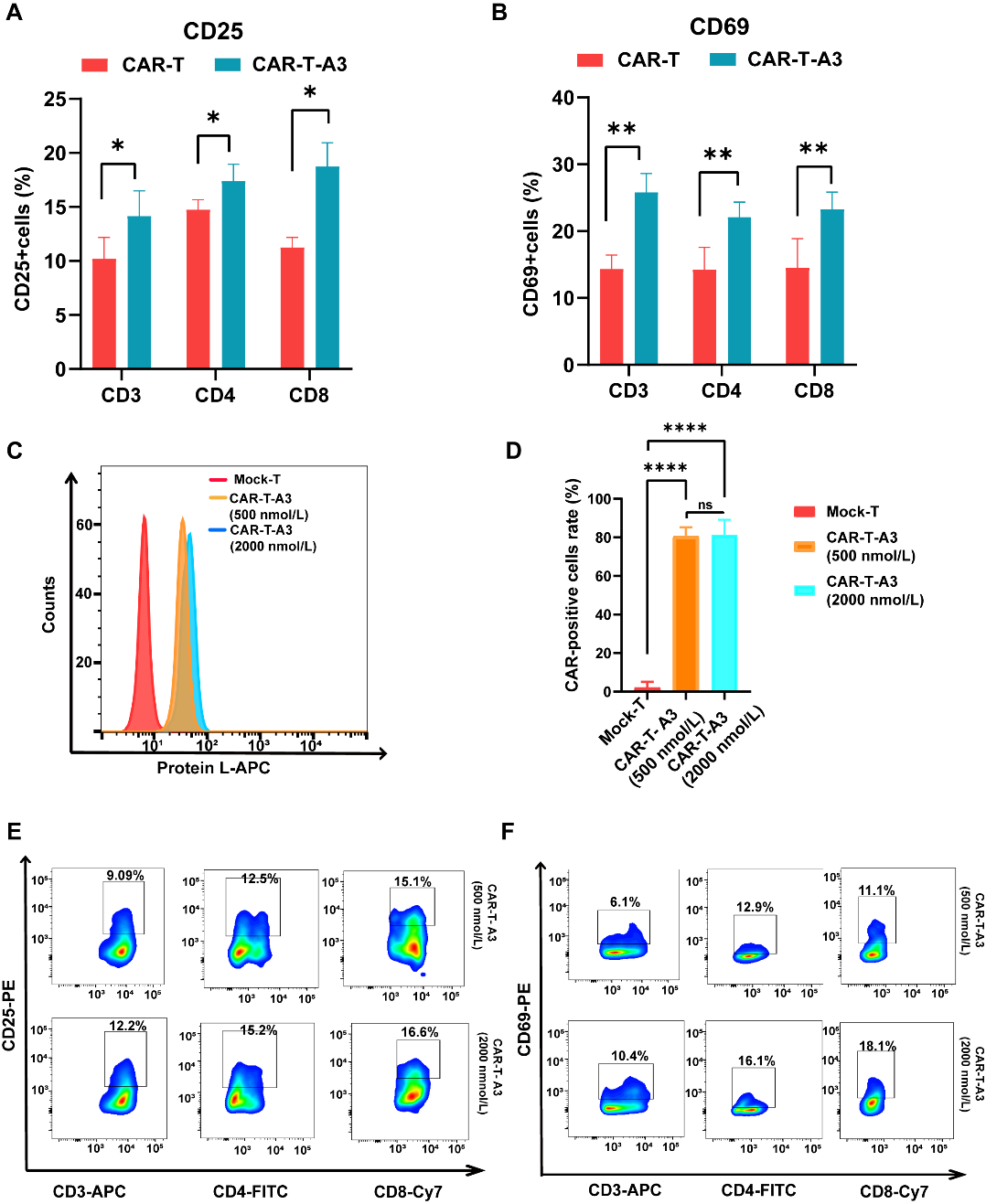


Figure S9. (A) Statistical graph of activation marker CD25 expression in gated CD3+, CD4+, and CD8+ T cell populations from CAR-T cells and CAR-T cells-Aptamer A3 (250 nmol/L). (B) Statistical graph of activation markers CD69 in gated CD3+, CD4+, and CD8+ T cell populations from CAR-T cells and CAR-T cells-Aptamer A3 (250 nmol/L). (C) CAR expression rates in CAR-T cells following their binding with varying concentrations of Aptamer A3 (500 nmol/L and 2000 nmol/L). (D) Statistical graph of the CAR-positive rate in CAR-T cells following their binding with varying concentrations of Aptamer A3 (500 nmol/L and 2000 nmol/L), defined as the percentage of T cells that were positive for protein L binding. (E-F) The expression of activation markers CD25 and CD69 in gated CD3+, CD4+, and CD8+ T cell populations from CAR-T cells- Aptamer A3. Error bars represented the standard deviation (n=3). *P < 0.05, **P<0.01, ***P<0.001, ****P<0.0001.


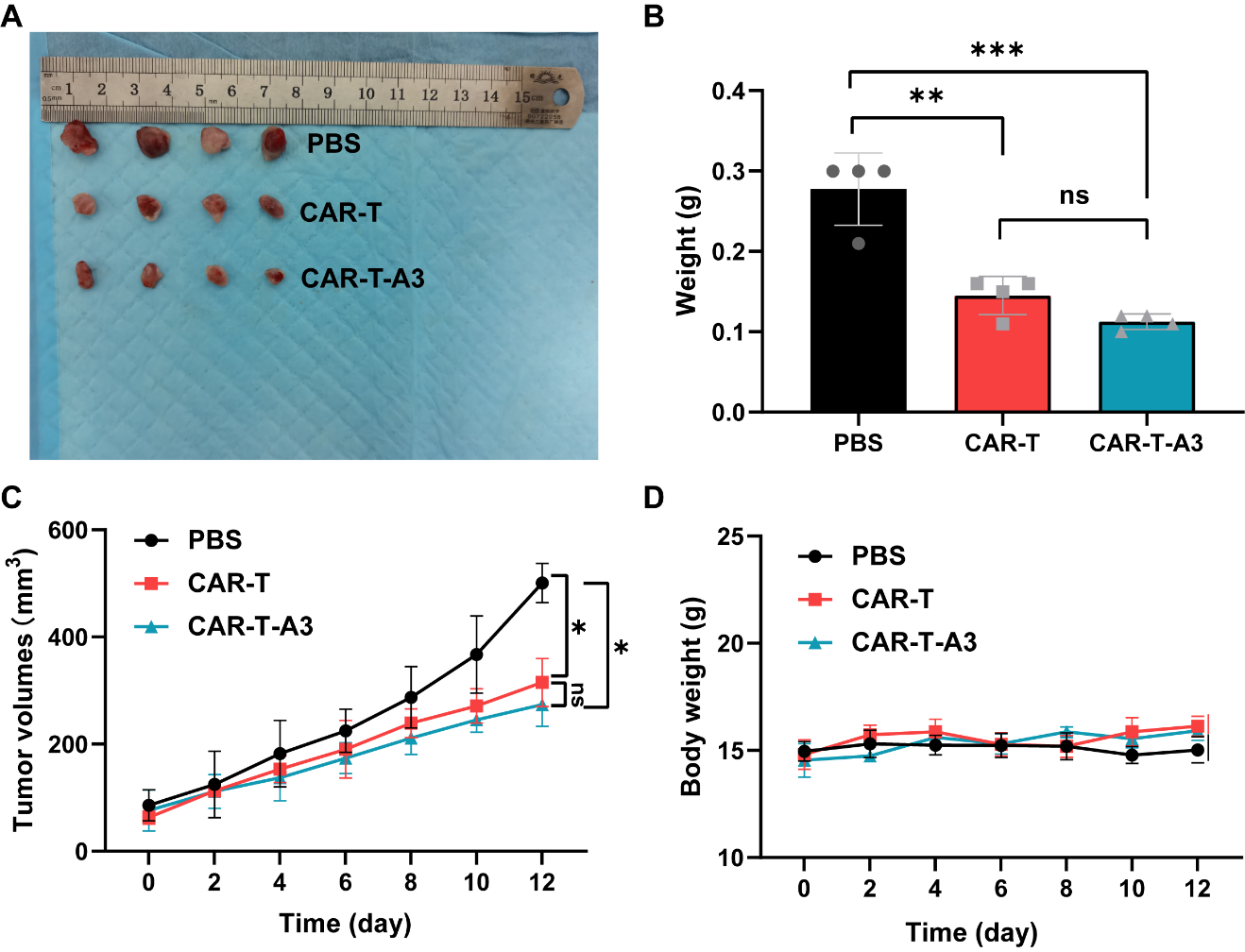


Figure S10. (A)The photographs of the ex vivo tumors after different treatments (PBS, CAR-T, CAR-T cells-Aptamer A3) for 12 days. (B) Tumor weights collected at day 12 after different treatments. (C) Tumor growth curves of different groups of mice after various treatments. (D) Body weights measurement in each group. Error bars represented the standard deviation (n=4), *P < 0.05.


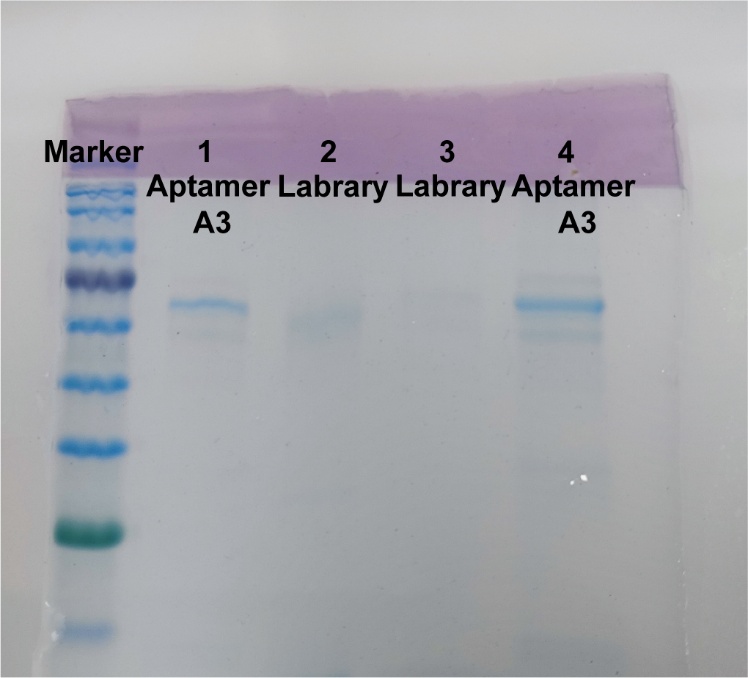


Figure S11. Full-size image of gel for Figure 3B. Coomassie Brilliant Blue stained 10 % SDS-PAGE was used to evaluate the protein binding to Aptamer A3.


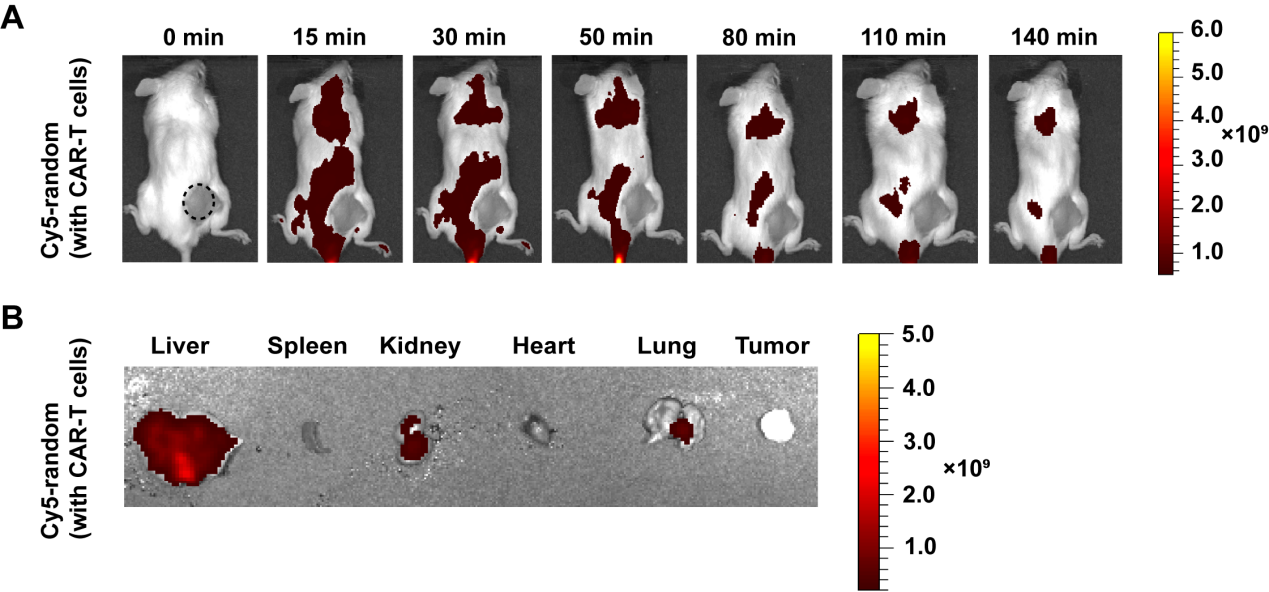


Figure S12. (A) Fluorescence images of model mice with tail vein injection of Cy5-labeled random library after CAR T cells. (B) Fluorescent images of liver, spleen, kidney, heart, lung and tumor of mice injected with Cy5-labeled random library.


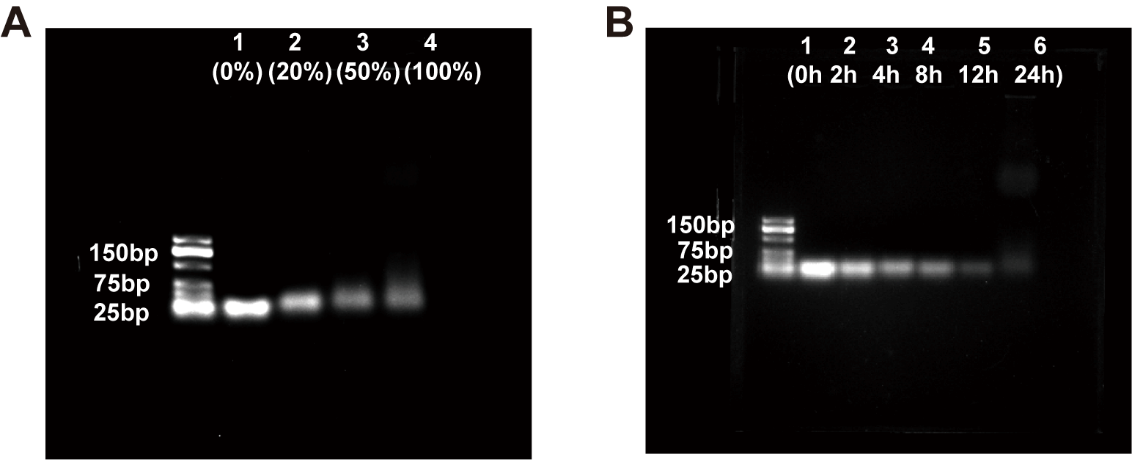


Figure S13. Full-size images of gel for Figure 4A-B. (A) Aptamer was in incubated different concentration of human serum for 2 hours. Lane 1: concentration of serum 0%; lane 2: 20%; lane 3: 50%; lane 4: 100%. (B) Aptamer was incubated different time in 20 % concentration of human serum. Lane 1:0 h; lane 2:2 h; lane 3:4 h; lane 4: 8 h; lane 5: 12 h; lane 6: 24 h.


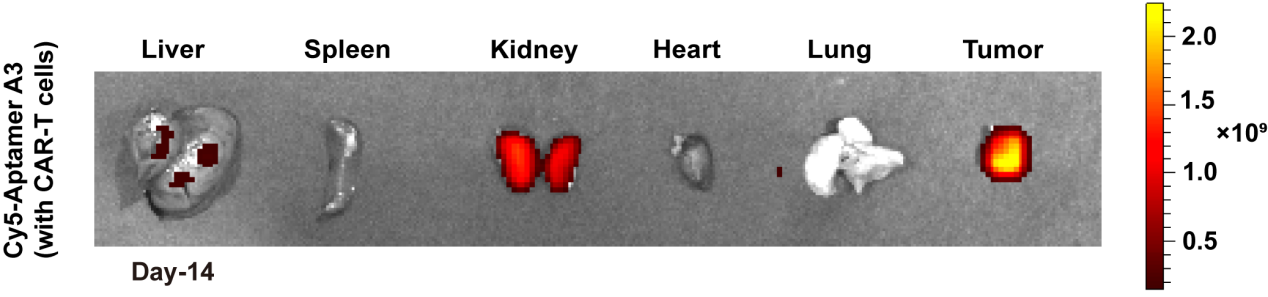


Figure S14. Fluorescent images of liver, spleen, kidney, heart, lung and tumor of mice injected with Cy5-labeled Aptamer A3 on day 14.


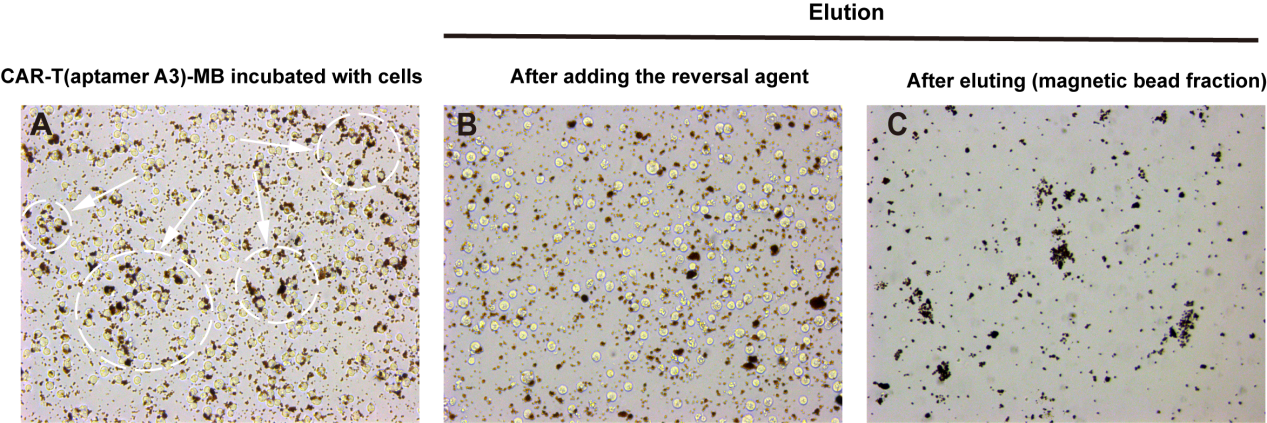


Figure S15. Microscopic observation of CAR-T cell binding to the magnetic beads during cell sorting. (A) Magnetic beads loaded with Aptamer A3 bind to CAR-positive T cells and form cell clusters (white circles and arrows) from the cell sample mixed by CAR-T cells and Mock-T cells. (B) CAR-positive T cells were released from magnetic beads after adding the reverse agent and the cell clusters disappeared. (C) CAR-positive T cells are eluted and completely separated from the magnetic bead fraction.

Table S1. Experimental conditions used in rounds of CAR-T cell SELEX.

| SELEX  Round | Positive  Selection | Negative  Selection | Aptamer  Pool(pmol) | BSA(%) | Washing  Strength | PCR Cycles |
| --- | --- | --- | --- | --- | --- | --- |
| 1 | 5 × 10^6^ | None | 2000 | 0.1 | 2min × 3 | None |
| 2 | 4 × 10^6^ | None | 400 | 0.1 | 2min × 3 | 14 |
| 3 | 4 × 10^6^ | None | 400 | 0.1 | 2min × 3 | 18 |
| 4 | 2 × 10^6^ | 1 × 10^6^ | 200 | 0.5 | 2min × 3 | 20 |
| 5 | 2 × 10^6^ | 1 × 10^6^ | 200 | 0.5 | 3min × 3 | 20 |
| 6 | 2 × 10^6^ | 1 × 10^6^ | 100 | 0.5 | 3min × 3 | 20 |
| 7 | 1× 10^6^ | 3 × 10^6^ | 100 | 0.5 | 3min × 3 | 22 |
| 8 | 1 × 10^6^ | 3 × 10^6^ | 80 | 1 | 3min× 4 | 21 |
| 9 | 1 × 10^6^ | 3 × 10^6^ | 80 | 1 | 3min× 4 | 27 |
| 10 | 0.5 × 10^6^ | 5 × 10^6^ | 50 | 1 | 3min× 4 | 22 |
| 11 | 0.5 × 10^6^ | 5 × 10^6^ | 50 | 1 | 3min× 4 | 25 |

Table S2. The detailed sequences of the selected aptamer candidates.

| Name | Center region(from5’to3’) | nt |
| --- | --- | --- |
| Aptamer A1 | ACCGCAGCGCACATCAACACGCATACCGTCACGTAG | 76 |
| Aptamer A2 | ACACCACACTGCACCTCGTCGTGACGCCATGCGCAC | 76 |
| Aptamer A3 | ACGCTGCCGTGTGCTGCATGTGGTCGTGTGTCCGTG | 76 |
| Aptamer A4 | CTGACCACCACCACACCACGACGTCCGATGAACGTG | 76 |
| Aptamer A5 | CACCACATCACGTCGAGCCACACGTCGGCCTGCGAT | 76 |
| Aptamer A6 | ACGTCACCACGATCTACGACAGCACACATGCGTTGA | 76 |
| Aptamer A7 | CTGCACCACCCTCGTCAACACATCGCACGTACCGTG | 76 |
| Aptamer A8 | CACGACGCGACGATGGCCGTACACTACCACGATGAT | 76 |

ssDNA library: TTCAGCACTCCACGCATAGC-N36-CCTATGCGTGCTACCGTGAA

Table S3. Sequences of aptamers and reversal agent used in experiments.

| Name | Sequences (5’-3’) | Modification |
| --- | --- | --- |
| Aptamer A3 | TTCAGCACTCCACGCATAGCACGCTGCCGTGTGCTGCATGTGGTCGTGTGTCCGTGCCTATGCGTGCTACCGTGAA | 5’biotin |
| Reversal agent | TTCACGGTAGCACGCATAGGCAC (23nt) |  |
| A3 sequences (after adding reversal agent) | TTCACGGTAGCACGCATAGGCACCTGCCGTGTGCTGCATGTGGTCGTGTGTCCGTGCCTATGCGTGCTACCGTGAA |  |
